# 3DReact: Geometric Deep Learning for Chemical Reactions

**DOI:** 10.1021/acs.jcim.4c00104

**Published:** 2024-07-15

**Authors:** Puck van Gerwen, Ksenia R. Briling, Charlotte Bunne, Vignesh Ram Somnath, Ruben Laplaza, Andreas Krause, Clemence Corminboeuf

**Affiliations:** † Laboratory for Computational Molecular Design, Institute of Chemical Sciences and Engineering, École Polytechnique Fédérale de Lausanne, 1015 Lausanne, Switzerland; ‡ National Center for Competence in Research − Catalysis (NCCR-Catalysis), École Polytechnique Fédérale de Lausanne, 1015 Lausanne, Switzerland; § Learning & Adaptive Systems Group, Department of Computer Science, ETH Zurich, 8092 Zurich, Switzerland

## Abstract

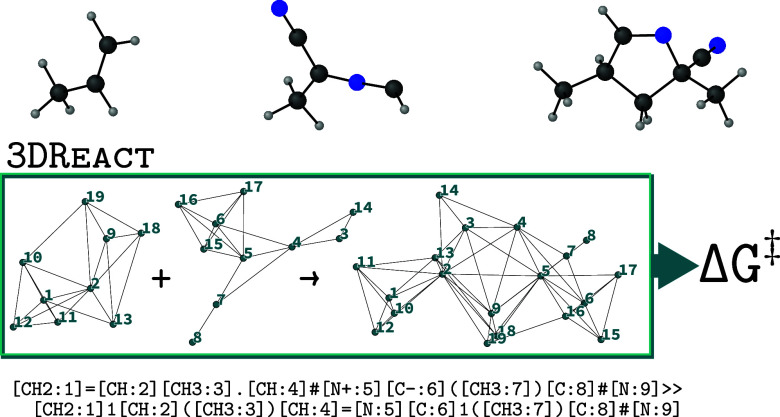

Geometric deep learning models, which incorporate the relevant molecular symmetries within the neural network architecture, have considerably improved the accuracy and data efficiency of predictions of molecular properties. Building on this success, we introduce 3DReact, a geometric deep learning model to predict reaction properties from three-dimensional structures of reactants and products. We demonstrate that the invariant version of the model is sufficient for existing reaction data sets. We illustrate its competitive performance on the prediction of activation barriers on the GDB7-22-TS, Cyclo-23-TS, and Proparg-21-TS data sets in different atom-mapping regimes. We show that, compared to existing models for reaction property prediction, 3DReact offers a flexible framework that exploits atom-mapping information, if available, as well as geometries of reactants and products (in an invariant or equivariant fashion). Accordingly, it performs systematically well across different data sets, atom-mapping regimes, as well as both interpolation and extrapolation tasks.

## 1 Introduction

Physics-inspired representations that take as input the three-dimensional structure^1−13^ (as well as, in some cases, electronic structure^14−17^) of molecules and transform it into a fixed-length vector, while respecting known physical laws, have a rich history in molecular property prediction.^1−10,12,13,18−30^ Common desiderata^31−34^ for high-performing representations are (i) smoothness, (ii) encoding of the appropriate symmetries to permutations, rotations and translations,^24,35^ (iii) completeness and (iv) additivity to allow for extrapolation to larger systems. Such fingerprints,^2−8,11−13,24^ being rooted in fundamental principles, are designed to be property-independent: a single representation can be constructed for a molecule to predict any quantum-chemical target. This is analogous to the molecular Hamiltonian, which specifies the energy and all other properties of a system as a function of atoms’ types and positions in three-dimensional space (assuming the molecules are charge neutral and singlets). These representations are typically used in combination with kernel models due to their data efficiency, ability to deal with high-dimensional feature vectors, and interpretability of the similarity kernel.^2−10,12,13,31−33^ Early works showed that combining such representations^2,4,6,8,36^ with simple feed-forward neural networks instead of kernel models did not necessarily led to better performance.^37,38^

More recently, end-to-end neural networks have been proposed that learn the representation as part of the (supervised) training process,^39−61^ based on similar principles to the aforementioned physics-inspired representations: they take as input a three-dimensional structure, as well as in some cases charge and spin information.^46,51−53^ The network may be *invariant* or *equivariant* to rotations and translations of the input molecules. The former is typically achieved by operating on distances between atoms,^39,40,42^ and the latter by operating on relative position vectors and angular information processed by rotationally equivariant convolutional layers.^41,43−46,48−50,54−59,62^ Equivariant models are naturally suited to predict vectorial^43−45,48,49,59,62^ or higher-order tensorial^52,54,55,59,63^ properties. They have also been demonstrated to exhibit improved data efficiency and generalization capabilities compared to their invariant counterparts on predictions of scalar properties,^43^ albeit at a higher computational cost. Nevertheless, given an expressive enough architecture (i.e., using higher-order messages^41,56,62,64−67^ and/or enough convolutional layers^43,52,56^), invariant models are sufficient for many property prediction tasks.^56^

Despite these advances for molecular property prediction, the prediction of computed *reaction properties* (principally, reaction barriers^36,38,68−82^) is still in its infancy.^83^ Machine learning approaches span from utilizing simple two-dimensional fingerprints of reaction components^84,85^ (reactants and products) to physical-organic descriptors,^75,76,80,82,86−98^ or electronic structure-inspired features,^99^ to transformer models^100,101^ adapted for regression,^102^ and 2D graph-based approaches.^70,71,81,103^ The latter, particularly the ChemProp model,^71,103^ are often best-in-class in predicting reaction properties.^103^ It has been shown^38^ that these models achieve their impressive performance by exploiting atom-mapping information,^104−107^ which provide information analogous to the reaction mechanism.

Another category of reaction fingerprints arises from discretization of physically inspired functions^2−10,12,13^ constructed using a cheap estimate of the transition state (TS) structure^73^ or rather the structures of the reaction components^36,69,74^ The SLATM_d_ representation^36,69^ in particular has been shown^38^ to yield accurate predictions of reaction barriers, particularly for data sets^69,108^ relying on subtle changes in the geometry of reactants and/or products. End-to-end models based on three-dimensional structures of reactants and products have also recently emerged.^72,99,109^ In a different vein, several works^110−115^ aim to directly predict the TS structure, which together with the reactant structure gives the reaction barrier. These approaches lie outside the scope of the property prediction focus here.

Due to the diversity of challenges posed by different reaction data sets, neither atom-mapping-based models nor 3D-geometry-based models achieve consistently better performance on reaction property prediction tasks.^38^ To date, no model has been proposed that can incorporate both chemical (atom-maps) and physical (geometry) priors. To address this gap, we introduce 3DReact, a geometric deep learning model that encodes both the three-dimensional structures of reactants and products as well as atom-mapping information or proxies thereof to predict properties of chemical reactions (showcased here for activation energies).

We demonstrate the performance of 3DReact on three data sets of reaction barriers: GDB7-22-TS,^116^ Cyclo-23-TS,^117^ and Proparg-21-TS.^69,108^ As discussed in previous works,^38^ these data sets present a myriad of challenges for ML models, from the dependence on chemical information^116^ to the distinction of subtle changes in configurations.^69,108^ We show that, compared to state-of-the-art models for reaction property prediction,^36,103^3DReact offers accurate and reliable performance across different data sets as well as atom-mapping regimes, reduced dependence on the quality of three-dimensional geometries, and stable extrapolation behavior.

## 2 Architecture

**Figure 1:**
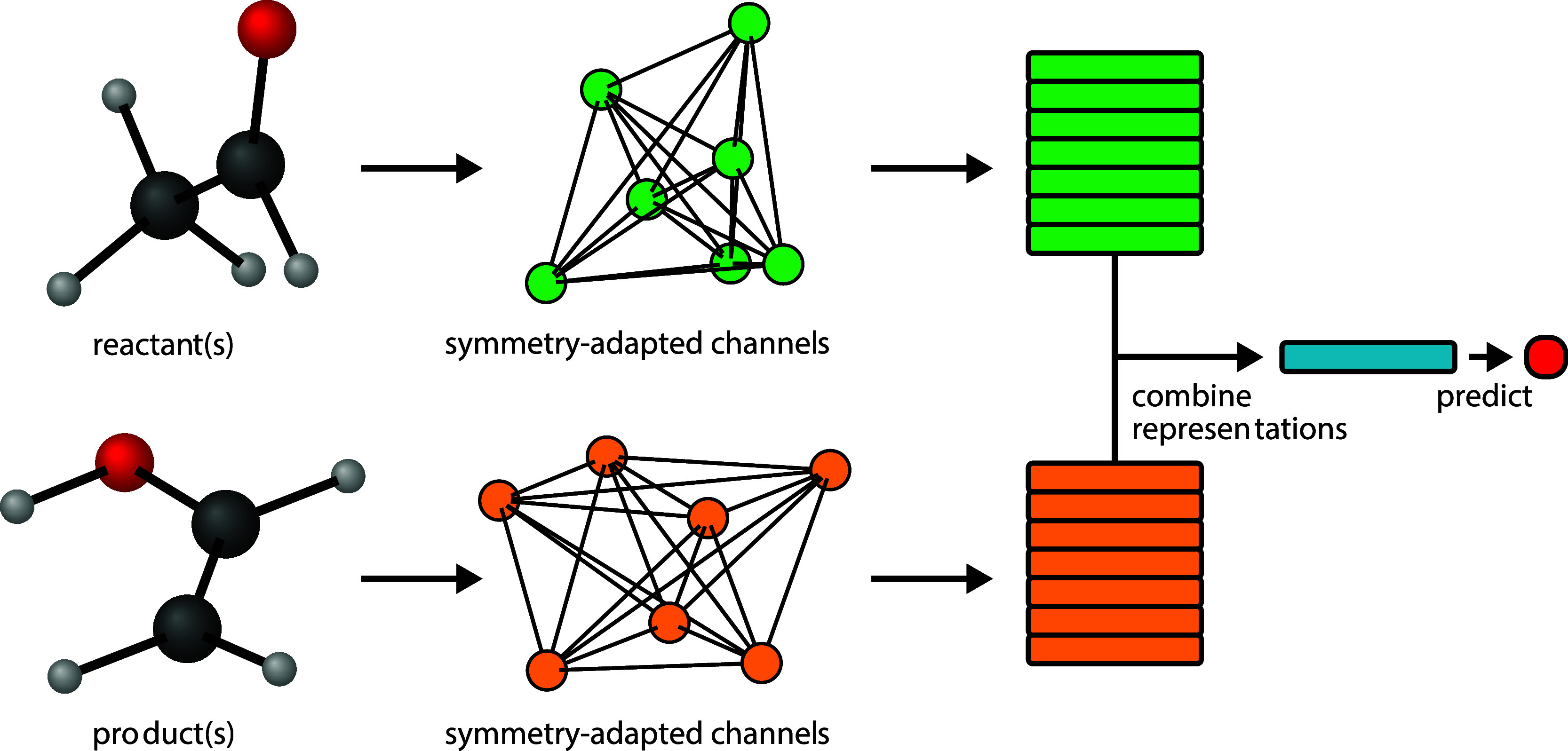
Architecture of 3DReact. Molecules pass through independent symmetry-adapted (invariant or equivariant) channels (green and orange). These are combined to yield a reaction representation (blue) which is used to predict a reaction property, such as the activation energy (red dot).

3DReact is built from O(3)-equivariant convolutional networks over point clouds as implemented in e3nn.^118^ Specifically, we use the tensor field network architecture^47^ for molecular components as in Corso et al.^119^ While the architecture is equivariant by default, it can easily be made invariant (vide infra). The geometries of molecules constituting reactants and products of each reaction are passed through separate channels, detailed in Section 2.1. They are then combined to eventually predict a reaction property, such as the activation energy, as detailed in Section 2.2. The overall architecture is summarized in Figure 1.

### 2.1 Symmetry-Adapted Molecular Channels

A molecule with *N*_at_ atoms is represented as a distance-based graph where nodes describe atoms and edges describe bonds. Instead of explicitly using connectivity information, the “bonds” of atom *a* are formed with all the neighboring Neigh(*a*) atoms within the cutoff *r*_max_. Initial scalar bond (edge) features {**e**_*ab*_^(0)^} between atoms *a* and *b*, as well as spherical harmonics filters {**z**_*ab*_}, are computed from internal coordinates, as detailed in eqs S1–S4. The atom (node) features {**x**_*a*_^(0)^} are initialized with *n*_*f*_ = 16 cheminformatics descriptors computed with RDKit.^120^ These include atomic number, chirality tag (unspecified, tetrahedral, or other, including octahedral, square planar, allene-type), number of directly bonded neighbors, number of rings, implicit valence, formal charge, number of attached hydrogens, number of unpaired electrons, hybridization, aromaticity, and presence in rings of specified sizes from 3 to 7. This choice is inspired by EquiBind(121) and DiffDock(119) and is in line with the improved^72^ features used for 2D-based methods.

The initial node and edge features pass through embeddings to give {**x**_*a*_^(1)^} and {**e**_*ab*_} respectively, the former are then updated by *n*_conv_ ∈ {2, 3} equivariant convolutional layers. Each layer is a fully connected weighted tensor product, as defined in e3nn.^118^Equations S5–S16 describe the equivariant operations performed by the network (see Section S1 for mathematical details). The network with equivariant molecular components as described is referred to as EquiReact, where its invariant counterpart InReact uses only the  = 0 (scalar) spherical harmonics to construct the convolution filters. The output of the molecular channels is the local molecular representation **X** ∈ ^*N*_at_×*D*^ corresponding to *N*_at_ atoms associated with *D* features. Depending on the sum_mode hyperparameter, it is constructed either from the node features (node mode) or both node and edge features (both mode).

Inspired by the ChemProp model,^71,103^ we added an option to exclude hydrogen atoms as nodes when constructing the graph. The only information about hydrogens is then contained in the initial edge features of heavy atoms.

### 2.2 Combining Molecules for Reactions

Once atom-wise molecular representations **X** are learned for reactant and product molecules, they must be combined to form a reaction representation **X**_rxn_.

For certain data sets, atom-mapping information is available, which correlates individual atoms in reactant molecules to individual atoms in product molecules according to the reaction mechanism. In this setting, the representations **X**_reactant_ and **X**_product_ are reordered such that the local representation vectors correspond to the same atom in reactants and products. Depending on the combine_mode hyperparameter, either a difference is taken between products’ and reactants’ atom representations, or they are summed, averaged, or passed through a multilayer perceptron (MLP). Thus, the local reaction representation **X**_rxn_ consists of vectors reflecting how the environment changes in the reaction for each atom. We will refer to this variant of the model, which uses atom-mapping information, as 3DReact_M_. While the current model is unable to treat unbalanced reactions (where there are additional atoms on the left- or right-hand side of the reaction equation), its modification in the spirit of ChemProp(71,103) is straightforward.

**Figure 2:**
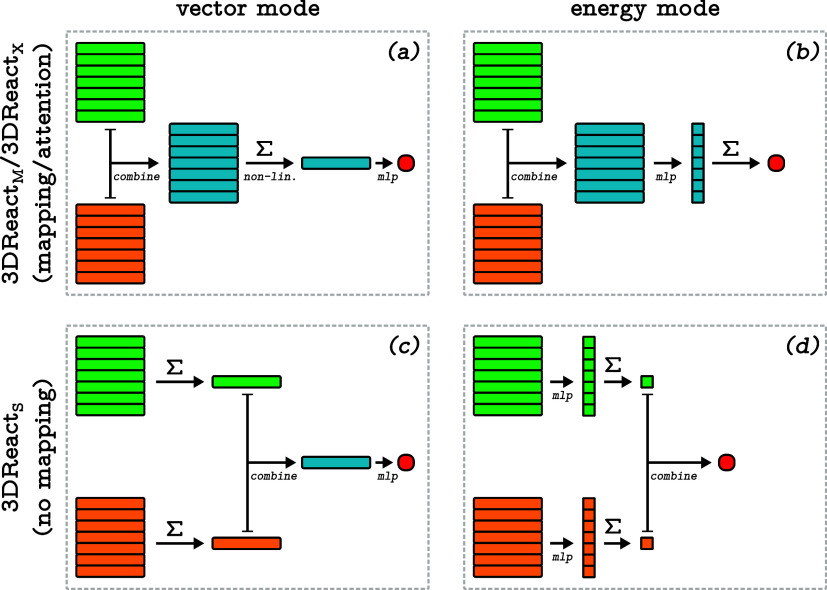
Scheme illustrating how the reactant (green) and product (orange) representations are combined to form a reaction representation (blue) and eventually predict the target property (red dot) using a multilayer perceptron (mlp). Σ refers to the summation over atom-wise environments. Oblong rectangles and squares represent vectors and scalars, respectively.

With the reaction representation at hand, predictions are made in the so-called vector or energy modes. In vector mode, the atomic vectors constituting the reaction representation **X**_rxn_ are initially passed through an MLP to introduce nonlinearity and then summed up to form a global reaction representation vector **X̅**_rxn_. The target is then learned using an MLP. This model pipeline is illustrated in Figure 2a. In energy mode, on the other hand, the local reaction representations are used to learn atomic contributions to the target (Figure 2b). While performing worse in general, in some cases this mode yields the best predictions (see Section 3.2.1).

Atom-mapping provides *static* information, analogous to a reaction mechanism, to link atoms in reactants to atoms in products. While highly informative, and thought to be critical to the performance of 2D-graph-based models,^70−72,81,103^ accurate atom-maps are not available for all reaction data sets.^38,104,105^ To circumvent the need for atom-mapping, but mimic its role in exchanging information between reactants and products, other approaches *dynamically* (i.e., in a learnable fashion) exchange information between molecular representations. For example, RXNMapper(107) is a neural network that learns atom-mappings within the larger self-supervised task of predicting the randomly masked parts in a reaction sequence, using one head of a multihead transformer architecture. EquiBind,^121^ a neural network that predicts the rotation and translation of a ligand to a protein, contains a cross-attention module between ligand and receptor. The latter inspires our surrogate for atom-mapping: 3DReact_X_ also uses cross-attention between reactants and products to link their atom indices (Section S4). The reordered representations of reactants and products are combined as for the case of atom-mapped reactions (Figure 2a,b). We note that other algorithms could also have been used to exchange information between reactants and products, for example in the form of message passing or equivariant attention.^57,122^

3DReact also has a simple “no mapping” variant, called 3DReact_S_, which does not rely on atom-mapping, nor a surrogate cross-attention module. In vector mode (Figure 2c), the atomic components of molecular representations **X**_reactant_ and **X**_product_ are summed up to obtain global vectors **X̅**_reactant_ and **X̅**_product_, respectively. Then they are combined, according to the combine_mode parameter, to form a reaction vector **X̅**_rxn_ which is used to learn the target with an MLP. In energy mode (Figure 2d) individual atomic representations are used to learn their contributions to the quasi-molecular energies of reactants and products, which are later combined (according to the combine_mode parameter) to predict the target. In most cases, this simpler model outperforms 3DReact_X_ (vide infra).

## 3 Results and Discussion

The performance of 3DReact is reported for three diverse data sets (the GDB7-22-TS,^116^ Cyclo-23-TS^117^ and Proparg-21-TS^69,108^) using both random and extrapolative splits. For details on the data sets, refer to Section 5.1. For details on the extrapolation splits, see Section 5.2.

Models are run in three atom-mapping regimes: (i) with high-quality maps (“True”) derived from the TS structures or heuristic rules;^71,106,116,117,123^ (ii) with atom-maps obtained using the open-source RXNMapper(107) (“RXNMapper”); and (iii) without any atom-mapping information at all (“None”). As discussed in recent work,^38,124^ previously developed graph-based models for reaction property prediction^70−72,96,97^ including ChemProp(71,103) reported prediction errors only in the “True” atom-mapping regime. The “RXNMapper” regime is important for cases where the reaction mechanism is not known and atom-mapping using heuristic rules is impossible. The “None” regime is critical for all chemistry that falls outside the realm of organic chemistry captured in the patents^125^ that RXNMapper(107) is trained on.

The atom-mapping-based model 3DReact_M_ is used in the “True” and “RXNMapper” regimes. In the “None” regime, 3DReact_X_ and 3DReact_S_ were tested. 3DReact_S_ consistently outperformed 3DReact_X_, so we include only 3DReact_S_ and refer the reader to Section S4 for their comparison.

### 3.1 Equivariance vs Invariance

**Figure 3:**
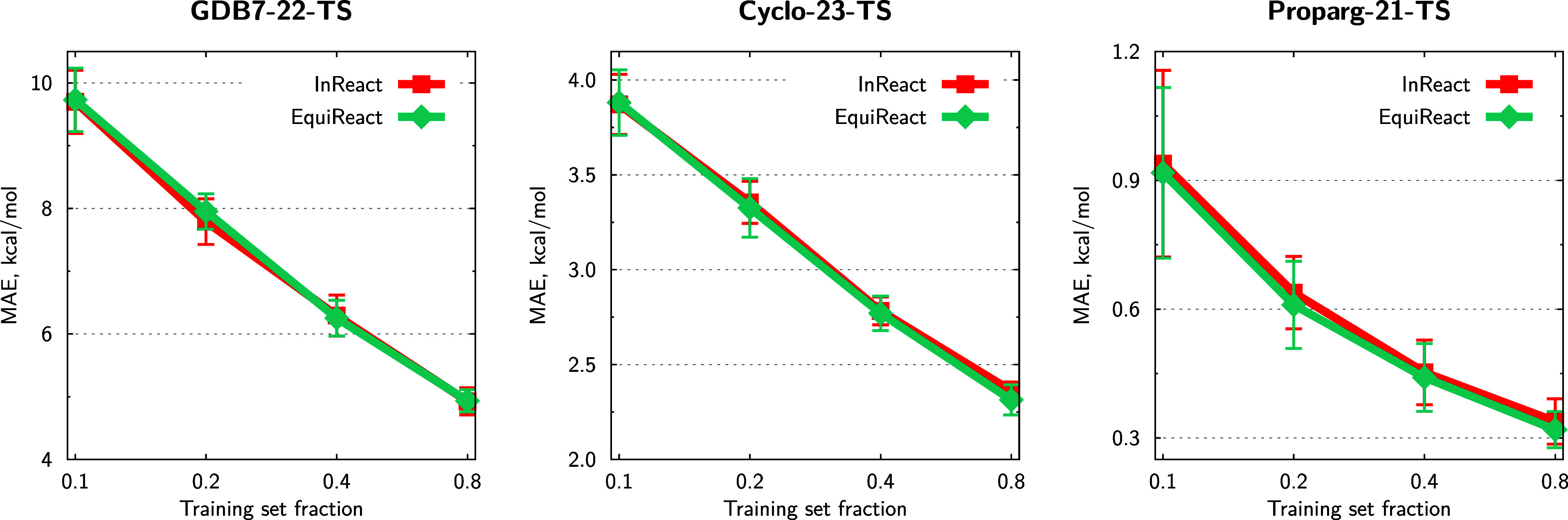
Learning curves for InReact and EquiReact in the “True” atom-mapping regime. Each point shows mean absolute error (MAE), averaged over 10 folds of 80/10/10 splits (for training set fraction < 0.8, the corresponding subset of the “full” training set is used), and error bars indicate standard deviations across folds.

Table 1 compares the relative performance of the invariant (InReact) and the equivariant (EquiReact) implementations of 3DReact with the learning curves of the two models presented in Figure 3. Previous studies^43,56^ demonstrated that the equivariant models showed superior extrapolation capabilities on predictions of energies and forces, as well as steeper and shifted learning curves in force prediction tasks. Instead, we find that InReact and EquiReact are practically indistinguishable for the present chemical reaction tasks.

**Table 1:** Performance as Measured in Mean Absolute Errors (MAEs) of Predictions of 3DReact (InReact vs EquiReact)^a^

Data set (property, units)	Atom-mapping regime	InReact	EquiReact
**Random Splits**			
GDB7-22-TS (Δ*E*^‡^, kcal/mol)	True	4.93 ± 0.18	4.93 ± 0.15
	RXNMapper	6.03 ± 0.26	6.05 ± 0.25
	None	6.56 ± 0.26	6.53 ± 0.28
Cyclo-23-TS (Δ*G*^‡^, kcal/mol)	True	2.39 ± 0.08	2.30 ± 0.09
	RXNMapper	2.37 ± 0.07	2.35 ± 0.12
	None	2.39 ± 0.05	2.31 ± 0.09
Proparg-21-TS (Δ*E*^‡^, kcal/mol)	True	0.33 ± 0.07	0.31 ± 0.05
	None	0.34 ± 0.06	0.31 ± 0.06
**Scaffold Splits**			
GDB7-22-TS (Δ*E*^‡^, kcal/mol)	True	7.8 ± 0.7	7.8 ± 0.8
	RXNMapper	9.2 ± 0.8	9.1 ± 0.8
	None	10.1 ± 0.9	10.0 ± 0.9
Cyclo-23-TS (Δ*G*^‡^, kcal/mol)	True	2.79 ± 0.18	2.72 ± 0.18
	RXNMapper	2.77 ± 0.22	2.71 ± 0.23
	None	2.76 ± 0.22	2.72 ± 0.19
Proparg-21-TS (Δ*E*^‡^, kcal/mol)	True	0.44 ± 0.11	0.40 ± 0.08
	None	0.45 ± 0.10	0.41 ± 0.09

a 3DReact_M_ is used for the “True” and “RXNMapper” regimes, and 3DReact_S_ is used for the “None” regime. MAEs are averaged over 10 folds of 80/10/10 splits (training/validation/test) and reported together with standard deviations across folds.

**Figure 4:**
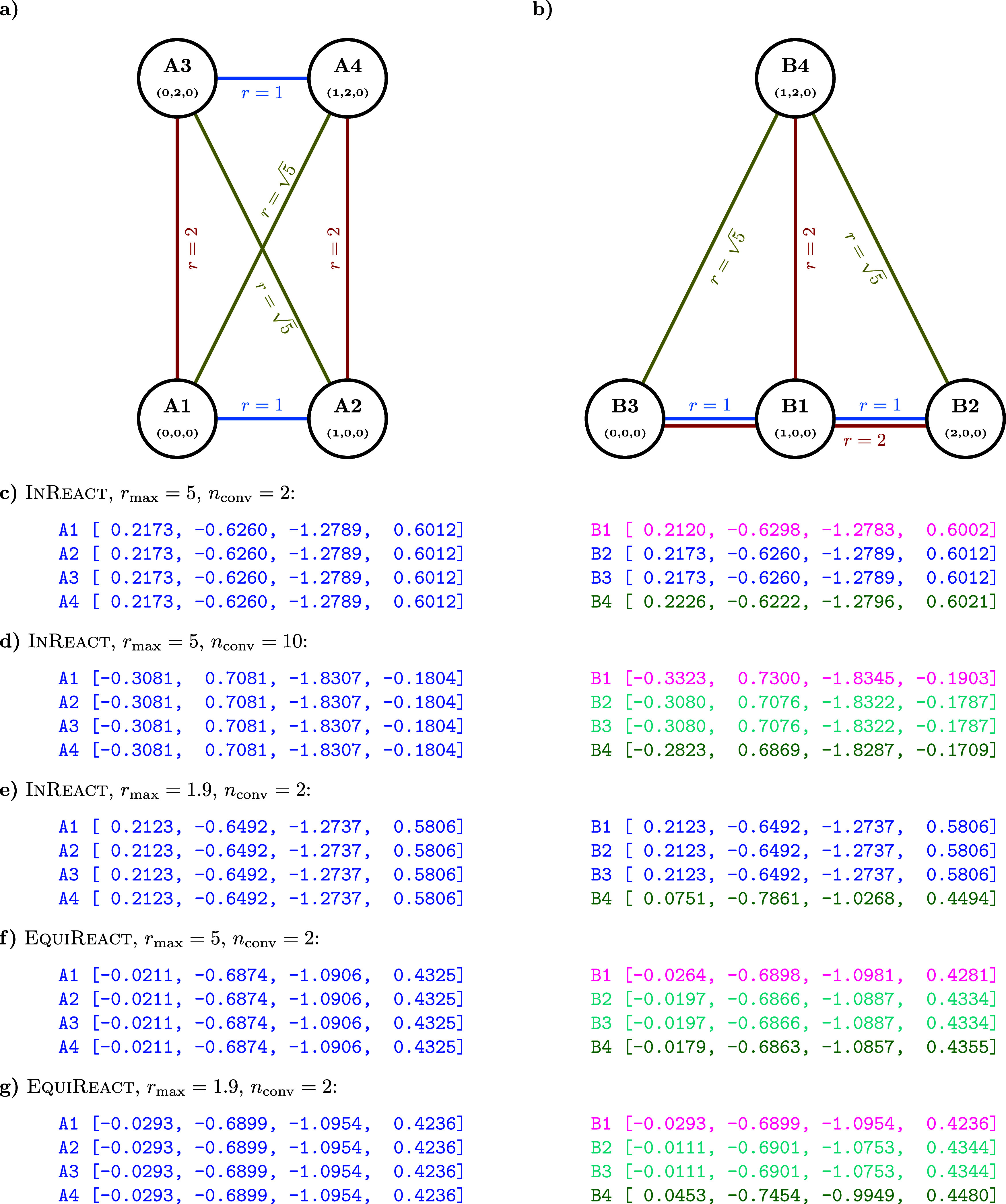
Top: Reactant and product of a toy reaction: two homometric structures (a) and (b) with atom labels, atom coordinates (Å), and interatomic distances (Å). “Bonds” of the same length are of the same color. Bottom: output after 5 epochs of the invariant (c,d,e)/equivariant (f,g) molecular channels for each atom with different radial cutoffs *r*_max_ and number of convolutional layers *n*_conv_. Within each subfigure, atomic representations indistinguishable up to shown digits are marked by the same color.

We find that the data sets studied herein do not benefit from the inclusion of equivariant features for molecules. Yet, Figure 4 illustrates that a hypothetical reaction involving conversion between homometric structures of He_4_,^126^ which is mostly characterized by angle changes, clearly benefits from equivariant molecular features. In the reactant (Figure 4a), all atoms are identical and lead to the same learned representation. In the product (Figure 4b), only atoms B2 and B3 have identical environments, different from A1–4. Atoms B2–3 have the same distances *r* to the three neighbors, as in A1–4. Thus, InReact, which uses only interatomic distances, yields very close representations for these atoms (Figure 4c). Still, in each convolutional layer, atoms B2 and B3 receive information from B1 and B4, and with increase of *n*_conv_ the difference in the representations of B2–3 and A1–A4 becomes more apparent (Figure 4d). However, with smaller radial cutoff *r*_max_ = 1.9, atoms B1–B3 and A1–4 become indistinguishable for any number of layers (Figure 4e). On the other hand, EquiReact, which uses explicit angular information from the spherical harmonics filters, clearly distinguishes all non-equivalent atoms in both cases already for *n*_conv_ = 2 (Figure 4f,g).

While this is a toy example, it illustrates that transformations consisting of changes in angles rather than in bond lengths are better described using EquiReact. In general, the currently available reaction data sets do not pose sufficient challenge to allow distinguishing InReact and EquiReact. For the data sets studied in this work, InReact is sufficient and is the model variation used throughout as 3DReact.

### 3.2 Benchmark Studies

3DReact is compared to previously best baseline models:^38^ChemProp,^71,103^ a graph neural network that uses atom-mapped SMILES to construct a CGR, and the 3D-structure-based SLATM^8^ fingerprint adapted to reactions by taking the difference between product and reactant fingerprints (SLATM_d_),^36^ combined with KRR models (SLATM_d_+KRR).

Note that both 3DReact and ChemProp are run without explicit H atoms, for two reasons. First, hydrogen atoms are not always mapped in the “True” and “RXNMapper” regimes, since they are usually implicit in SMILES strings. Second, there is no consistent improvement in including H atoms in the models (Table S6). SLATM_d_, built directly from molecular coordinates without using SMILES strings, does however incorporate H atoms by default. For further discussion refer to Section S7.

#### 3.2.1 Random Splits

Performance as measured in mean absolute errors (MAEs) is illustrated in Table 2 for random splits of each data set, demonstrating the models’ interpolative capabilities. For the equivalent results with root mean squared errors (RMSEs), consult Section S3.

**Table 2:** Performance as Measured in Mean Absolute Errors (MAEs) of Predictions of 3DReact vs State-of-the-Art Baselines ChemProp and SLATM_d_+KRR^a^

Data set (property, units)	Atom-mapping regime	ChemProp	SLATM_d_+KRR	3DReact
GDB7-22-TS (Δ*E*^‡^, kcal/mol)	True	**4.35** ± **0.15**		4.93 ± 0.18
	RXNMapper	**5.69** ± **0.17**		**6.03** ± **0.26**
	None	9.04 ± 0.21	**6.89** ± **0.20**	**6.56** ± **0.26**
Cyclo-23-TS (Δ*G*^‡^, kcal/mol)	True	2.69 ± 0.10		**2.39** ± **0.08**
	RXNMapper	2.71 ± 0.07		**2.37** ± **0.07**
	None	2.71 ± 0.12	2.65 ± 0.08	**2.39** ± **0.05**
Proparg-21-TS (Δ*E*^‡^, kcal/mol)	True	1.53 ± 0.14		**0.33** ± **0.07**
	None	1.56 ± 0.16	**0.33** ± **0.04**	**0.34** ± **0.06**

a All datasets are compared in three atom-mapping regimes: “True”, “RXNMapper” and “None”, except for the Proparg-21-TS set, where RXNMapper cannot map the reaction SMILES. MAEs are averaged over 10 folds of random 80/10/10 splits (training/validation/test) and reported together with standard deviations across folds. The lowest errors for each regime and dataset are highlighted in bold, if statistically relevant.

The GDB7-22-TS data set is distinct from the other two in that it includes variations in the reaction class (and mechanism), thereby showing a greater dependence on the existence and quality of atom-mapping information in the models. It has already been observed^38^ for ChemProp that there is stark hierarchy in the predictions from the “True” to “RXNMapper” to “None” regimes.

In the “True” regime, 3DReact does not improve predictive capabilities over the ChemProp model for the GDB7-22-TS set. This points to the importance of the chemical diversity in this data set, where knowledge of the reaction mechanism (in the form of atom-maps) is sufficient information to predict the reaction barriers without information about the geometries of reactants and products. However, as previously discussed,^38^ “True” maps are an unrealistic scenario for most data sets. Moving to the “RXNMapper” regime, 3DReact and ChemProp already agree within standard deviations. This highlights that for practical-quality maps, 3DReact is among the best models for this data set. In the “None” regime, 3DReact outperforms ChemProp by more than 2 kcal/mol.

SLATM_d_+KRR results in similar performance to 3DReact for the GDB7-22-TS set. The SLATM_d_ representation also constructs features from 3D coordinates of the reactants and products using invariant functions, and is therefore more fundamentally similar to 3DReact than ChemProp. Nevertheless, since 3DReact allows for the inclusion of atom-mapping information, predictions are improved in the mapped regimes compared to SLATM_d_+KRR, which operates in the “None” regime only.

In summary, for the chemically diverse GDB7-22-TS set, while SLATM_d_ allows for good performance in the “None” regime, and ChemProp in the “True” and “RXNMapper” regimes, since 3DReact can incorporate both atom-mapping information and 3D structure information, the model achieves robust performance in all three regimes, with the predicted MAEs ranging from 4.93 to 6.56 kcal/mol.

The Cyclo-23-TS^117^ data set contains a single reaction class and has been previously illustrated^38^ to show less dependence on the quality of atom-mapping than the GDB7-22-TS. For this set, 3DReact outperforms or matches the other models in all three regimes. This illustrates that a model based purely on geometry information of reactants and products, without any chemical information in the form of atom-mapping or surrogates thereof, can allow for accurate reaction property prediction. It is worth noting that atom-mapping does not improve predictions at all, i.e., there is no improvement from “None” to “RXNMapper” to “True”, even for the ChemProp model. This points to the different nature of this data set compared to the GDB7-22-TS.

The best model is obtained with 3DReact_S_ in the energy mode (Figure 2d). As outlined in Section 2.2, in energy mode an energy contribution is learned for reactants’ and products’ atoms separately. In the original publication,^117^ Stuyver et al. illustrate that the activation barriers (Δ*G*^‡^) correlate linearly with the reaction energy (Δ*G*). Since Δ*G* is the difference between products’ and reactants’ energies, the energy mode is the best choice for a model learning the reaction energy, and in the case of this data set, for Δ*G*^‡^ too, due to its linear correlation with Δ*G*.

Compared to SLATM_d_+KRR, 3DReact in the “None” regime results in lower prediction errors for this set, illustrating that despite both models using similar information, an end-to-end model can allow for improved predictions.

The Proparg-21-TS^69,108^ is a small data set for neural network standards (753 points) and therefore constitutes a challenge for the data efficiency of our model. Like the Cyclo-23-TS set, it consists of a single reaction class, i.e., enantioselective propargylation of benzaldehyde. Since the enantioselectivity is related to the barrier through an exponential relationship, it is critical to predict the barrier accurately (≤1 kcal/mol).^69^ The “RXNMapper” regime is not available since RXNMapper cannot atom-map the reaction SMILES of this set.

In the other regimes, 3D-structure-based models lead to the best results, outperforming ChemProp by a large margin. Proparg-21-TS is particularly hard for 2D-based models^38^ since it contains molecules of different stereochemistry but the same SMILES strings. Again trained on a single reaction class data set, models do not benefit from being provided the “obvious” chemical information: including true atom-maps does not decrease the error. Competing only in the “None” regime, 3DReact does not allow for a performance improvement compared to SLATM_d_+KRR. Given the small size of the data set, it is already a demonstration of data efficiency that the deep-learning model matches the prediction errors of the kernel model. Unlike for Nequip(43) however, the data efficiency here is not due to the equivariant molecular components (Section 3.1).

The three data sets illustrate the benefits of the flexibility of 3DReact: depending on the data sets’ particular challenges, the model exploits the available information to yield the best-performing model in almost all cases. Since the model settings (such as vector or energy mode choice) are hyperparameters, the optimized version of 3DReact can emerge with minimal user intervention.

#### 3.2.2 Extrapolative Splits

**Figure 5:**
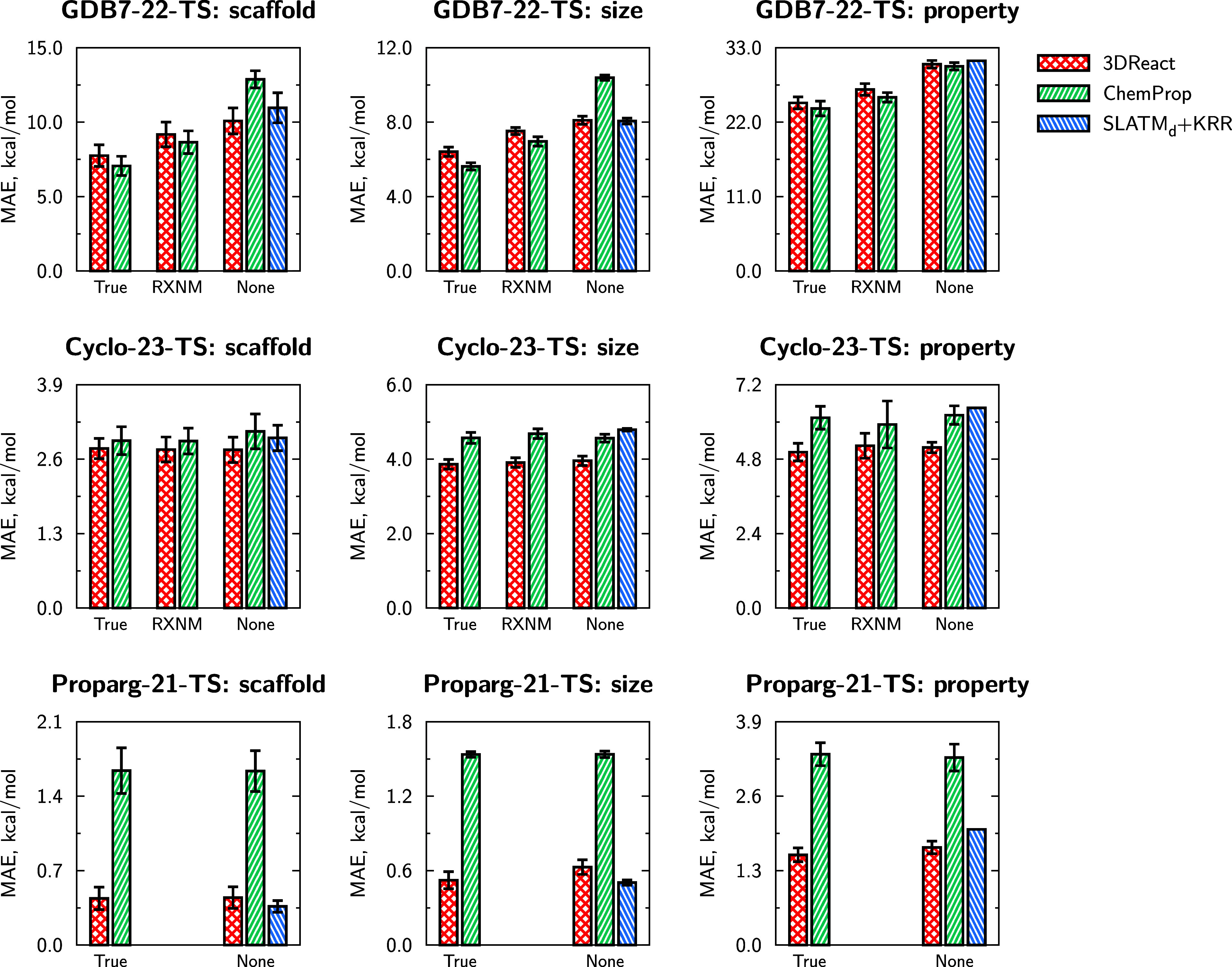
Mean absolute errors (MAEs) of predictions using three different extrapolation splits: scaffold, size-, and property-based. All data sets are compared in three atom-mapping regimes: “True”, “RXNMapper” (RXNM), and “None”, except for the Proparg-21-TS set, where RXNMapper cannot map the reaction SMILES. MAEs are averaged over 10 folds of 80/10/10 splits (training/validation/test), and error bars indicate standard deviations across folds, where applicable.

Figure 5 illustrates model performance for extrapolative splits (based on scaffolds, molecular size of reactants/products, and barrier magnitude, detailed in Section 5.2). Table S5 shows results of additional experiments, including comparing invariant and equivariant versions of 3DReact. These different types of extrapolative splits are necessarily more difficult than random splits, as demonstrated by higher MAEs in Figure 5. The relative performance of the models is largely maintained in the three different extrapolation regimes compared to the interpolation regime presented in Table 2.

Bemis–Murcko scaffold^127^ splitting clusters molecules (reactants for GDB7-22-TS and Proparg-21-TS, products for Cyclo-23-TS) based on ring systems. Test molecules may therefore appear “novel” from the point of view of the reaction graph, but will still feature distances and angles close to what the model has seen during training. Similarly for size-based splits, since there is no correlation between reactant/product size and reaction barriers, using distance information allows for stable predictions on extrapolation. Property-based splits are more challenging than the other two. For the Cyclo-23-TS and Proparg-21-TS sets, 3DReact still offers respectable errors, lower than those of the other models. For the GDB7-22-TS set, however, all models result in unreasonable MAEs over 20 kcal/mol. This points to the particular challenges of the GDB7-22-TS set and suggests an avenue for further developments of ML models for extrapolative tasks.^81^

Again in contrast to previous works that suggested equivariant models might be better at extrapolation tasks,^43,56^ here we find that 3DReact offers stable extrapolation performance (particularly for size- and scaffold-based splits), but not necessarily improved extrapolation behavior compared to 2D-graph-based models. This points to the different challenges in reaction property prediction. Nevertheless, Figure 5 illustrates that 3DReact is a consistently robust model for the three data sets when moving from interpolation to extrapolation regimes.

### 3.3 Model Behavior

Since the GDB7-22-TS set has the largest chemical diversity amongst the data sets explored, studying 3DReact and baseline models SLATM_d_ and ChemProp on this data set best captures the different chemical interpretation provided by these models.

**Figure 6:**
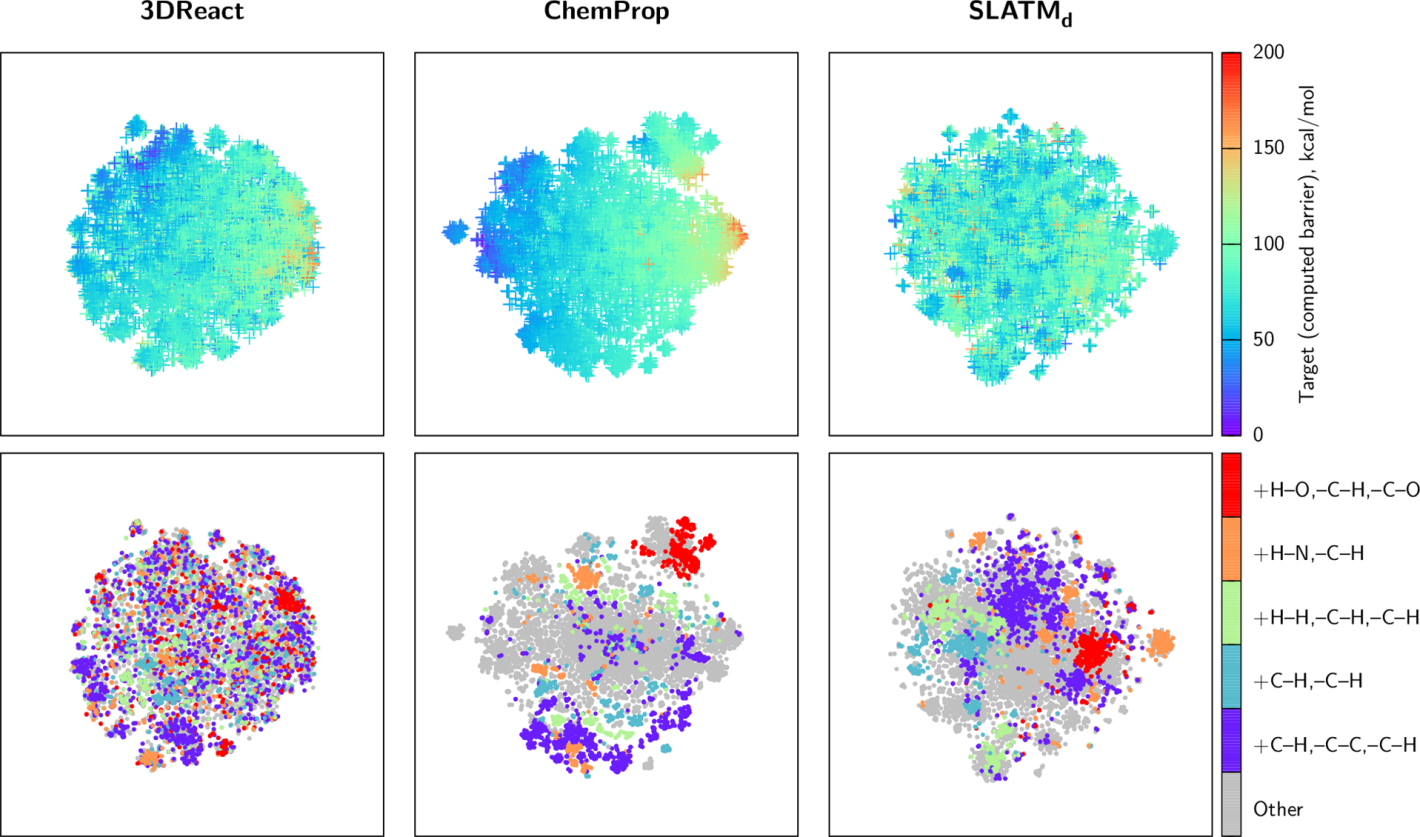
t-SNE maps (perplexity = 64) of the latent representations of 3DReact and ChemProp models in the “True” regime and the SLATM_d_ representation of the GDB7-22-TS data set, colored by the target Δ*E*^‡^ (upper panels) and reaction types (lower panels).

Figure 6 compares the (latent) representations of 3DReact “True”, ChemProp “True” and SLATM_d_ using t-SNE^128^ maps. In the upper panel, we find that the quality of the correlation between the representations and the target property are aligned with the relative performance of the models (Table 2). ChemProp and 3DReact show a smooth transition of the target property, whereas the map of SLATM_d_ does not have a clear structure. The lower panel shows the correlation of the representations with the five most common reaction types defined by bond breaking and formation (see Section 5.4). ChemProp, as a chemically inspired model, illustrates clear clusters in the reaction type. While SLATM_d_ is a geometry-based model, the binning structure used to create the representation^8,36^ results in a clear correlation with the reaction types, since e.g., the pairwise bins naturally cluster features such as C–H bond formation or breaking. 3DReact shows the least distinct “chemical” clustering, due to the interplay of geometry and mapping information exploited in the representation.

**Figure 7:**
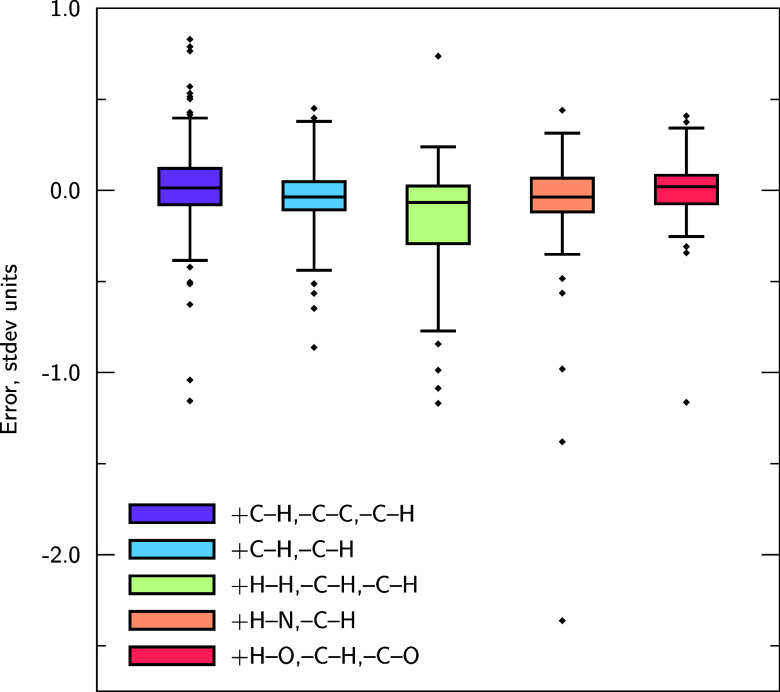
Box plots illustrating how 3DReact “True” performs for the most common reaction types in the GDB7-22-TS set. 3DReact is constructed without explicit H nodes in the graphs. The boxes range from the first to the third quartile of the data points. The whiskers limit 90% of the data points and the individual points illustrate outliers. The points correspond to the test set of the first random split. The errors are given in the target standard deviation (stdev) units (21.8 kcal/mol).

Figure 7 shows the error distribution of predictions belonging to the same reaction classes for 3DReact “True”. 3DReact performs universally well across the different reaction types, with consistently low errors and relatively small error spread. The reactions for which the model has higher mean errors and spread (+H–H,–C–H,–C–H (green)) correspond to those involving C–H and H–H features. Since the model is trained without explicit H nodes in the graph, features associated with X–H bonds are included implicitly in the model. Capturing H–H bond changes will be the most challenging as these will be the least explicitly described, occurring only as initial features for neighboring nodes. Since C is the most frequently occurring element in various different configurations, capturing all the C–H features is more challenging than the O–H features for example, which will be more similar to one another. The equivalent plot for the model trained with explicit H nodes is shown in Figure S4, illustrating that the error spread reduces for the reaction types involving C–H and H–H features. Note that 3DReact without explicit Hs still leads to performance comparable to the variant with explicit Hs (Section S7).

### 3.4 Geometry Quality

**Figure 8:**
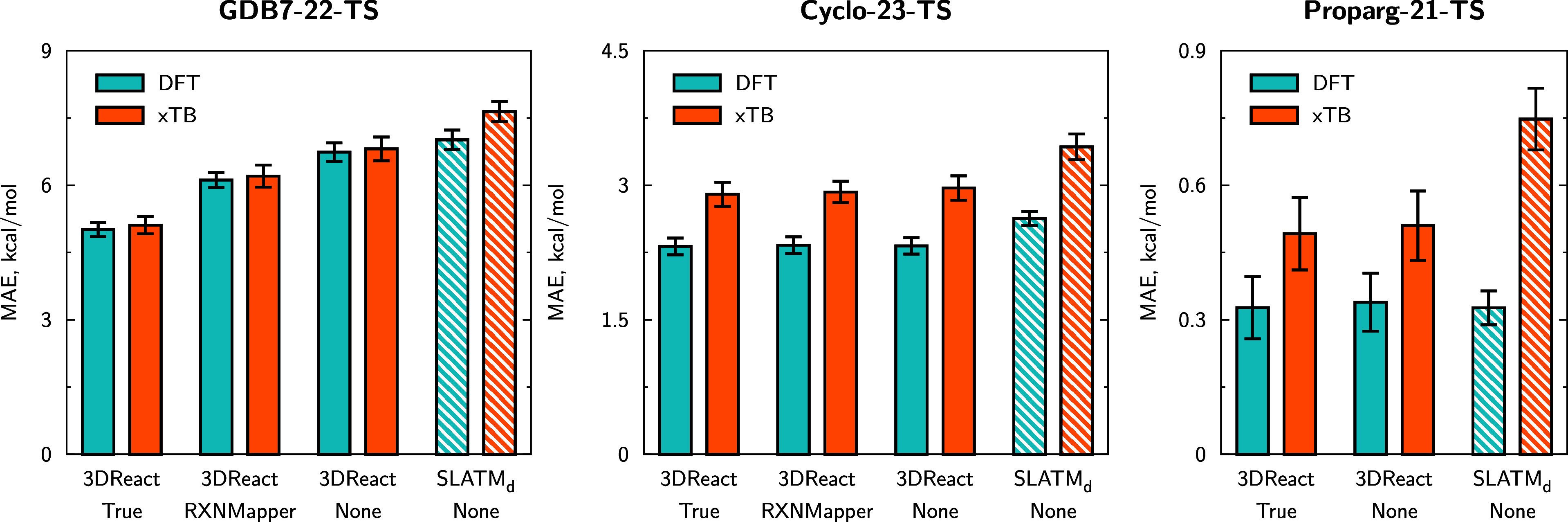
Mean absolute errors (MAEs) for predictions using either the provided geometries (ωB97X-D3/def2-TZVP for GDB7-22-TS, B3LYP-D3(BJ)/def2-SVP for Cyclo-23-TS, B97D/TZV(2p,2d) for Proparg-21-TS) (DFT) or lower-quality GFN2-xTB (xTB) geometries. MAEs are averaged over 10 folds of random 80/10/10 splits (training/validation/test), error bars showing standard deviations across folds. Note that for GDB7-22-TS and Cyclo-23-TS data sets, the DFT results are different from those presented in Section 3.2.1 because here they are obtained on the same subset as the xTB results (see Section 5.4).

In order to illustrate that 3DReact does not require high-quality molecular structures to be used in an out-of-sample scenario, we train and test a model using lower-quality GFN2-xTB^129^ (xTB) geometries to predict higher-level barriers (CCSD(T)-F12a/cc-pVDZ-F12//ωB97X-D3/def2-TZVP for GDB7-22-TS, B3LYP-D3(BJ)/def2-TZVP//B3LYP-D3(BJ)/def2-SVP for Cyclo-23-TS and B97D/TZV(2p,2d) for Proparg-21-TS). The results are illustrated in Figure 8 for the three data sets with DFT and xTB geometries, and compared to the SLATM_d_+KRR model in the same settings. 3DReact benefits from a lower sensitivity to the geometry quality compared to the predesigned representation SLATM_d_ combined with KRR, across the three data sets.

For the GDB7-22-TS set, there is a negligible difference in model performance moving from DFT to xTB geometries. The xTB geometries are a good proxy for the DFT ones here, since this set consists of small, charge-neutral organic molecules, which are largely well-described by semiempirical methods. For the Cyclo-23-TS set, while the molecules are still organic, they are larger than those in the GDB7-22-TS set, and there is a greater divergence between the GFN2-xTB and DFT geometries, resulting in a larger deterioration with these structures. Figure S5 demonstrates that when using the model trained on xTB geometries, barrier predictions for molecules with poorer geometries (i.e., higher RMSD of xTB vs DFT geometries) are not necessarily worse than those on molecules with better geometries. Instead, there is a consistent decline in model performance when training with xTB geometries and predicting DFT barriers.

The Proparg-21-TS set is the most complex of the three for GFN2-xTB, since these systems with charged organosilicon compounds differ considerably from those used to parametrize semiempirical methods or force fields. As described in Section 5.4, unlike for the other data sets where we generate an initial structure from SMILES using force fields, for this set it is impossible and we instead generate xTB geometries from the DFT ones. While this is not a feasible geometry generation pipeline for out-of-sample predictions, it still demonstrates how different methods perform with high and low-quality geometries. Here, we see that 3DReact is less sensitive than SLATM_d_+KRR and the variation trained with lower-quality geometries still offers competitive errors (0.48 ± 0.05 kcal/mol for the “None” model).

## 4 Conclusions

The accurate and reliable prediction of reaction barriers across diverse sets of chemical reactions remains an open challenge in computational chemistry. We contribute to this domain by introducing 3DReact, a geometric deep learning model constructed from the 3D coordinates of reactants and products. We show that the invariant model (vs the equivariant version) is already sufficient for currently available reaction data sets. Existing models ChemProp and SLATM_d_+KRR exhibit impressive performance for atom-mapped, chemically diverse data sets and stereochemistry-sensitive data sets, respectively. 3DReact offers a hybrid model that can optionally incorporate mapping information alongside geometries, enabling robust performance across different data set types and atom-mapping regimes. 3DReact also allows for a reduced sensitivity to the training geometry quality (i.e., xTB vs DFT level) compared to SLATM_d_+KRR. Predictions are stable both when moving to molecular size- or scaffold-based splits. Altogether, 3DReact presents a flexible framework for accurate prediction of activation barriers across chemical reaction data sets. Despite the proposed developments, challenges remain for ML predictions of energy barriers, particularly in integrating them within experimental settings. This work is a step toward their reliable application.

## 5 Methods

### 5.1 Data Sets

We test 3DReact on three data sets of reaction barriers previously used to benchmark reaction representations.^38^ The term “reaction barrier”, used interchangeably with “activation energy” and “activation barrier” is the energy difference between the energy of the optimized TS and the optimized reactants. Note that depending on the data set, some provide purely electronic energies (labeled Δ*E*^‡^) and others — Gibbs free energies (labeled Δ*G*^‡^). In all data sets, optimized three-dimensional structures of reactants and products are provided, which are used to train models and make predictions. The activation barrier is not a direct function of these structures, but using the TS structure to make predictions removes the utility of the ML models vs direct computation of the TS. Thus, we use an implicit interpolation of reactants’ and products’ structures as a proxy for the TS as in previous works.^36,38,69^

The GDB7-22-TS^116^ data set consists of close to 12 000 diverse organic reactions automatically constructed from the GDB7 data set^130−132^ using the growing string method^133^ along with corresponding energy barriers (Δ*E*^‡^) computed at the CCSD(T)-F12a/cc-pVDZ-F12//ωB97X-D3/def2-TZVP level. The data set provides atom-mapped SMILES, with “True” maps derived from the transition state. For 43 reactions out of 11 926, one of the products’ SMILES represents a molecule different from the xyz structure. These reactions were therefore excluded from the data set, leading to a modified GDB7-22-TS set used here.

While there are no predefined classes for all the reactions in the GDB7-20-TS^123^ or GDB7-22-TS^116^ sets, Grambow et al.^70^ split the data set into reactions undergoing certain bond changes: for example, the most common type was breaking of a C–H bond (−C–H) and a C–C bond (−C–C) in the reactants and formation of a C–H bond (+C–H) in the products, giving the reaction type signature +C–H,–C–C,–C–H. Here, we extract similar reaction types by comparing the connectivity matrices from atom-mapped reaction SMILES of reactants and products (ignoring bond orders). The most abundant reaction types in the data set are +C–H,–C–C,–C–H (1667 reactions), +H–N,–C–H (633), +C–H,–C–H (619), +H–O,–C–H,–C–O (599) and +H–H,–C–H,–C–H (517).

The original Cyclo-23-TS^117^ data set encompasses 5269 profiles for [3 + 2] cycloaddition reactions with activation free energies (Δ*G*^‡^) computed at the B3LYP-D3(BJ)/def2-TZVP//B3LYP-D3(BJ)/def2-SVP level in water using the SMD continuum solvation model. The data set provides atom-mapped SMILES with “True” maps for heavy atoms derived from either the transition state structure or heuristic rules. For the regime with explicit hydrogen atoms, we atom-mapped the xyz files by matching the reactants, given in two separate files, to the provided transition state structure, which closely resembles the two reactants and has the same atom order as in the product. This was done with a labeled graph matching algorithm as implemented in NetworkX.^134,135^ The algorithm is unaware of chirality, double-bond stereochemistry or conformations, and thus may lead to not exactly correct atom-mappings. We also found that in four reactions, the product SMILES and xyz files depict different species. Thus, the set was reduced to 5265 reactions.

The Proparg-21-TS data set^69,108^ contains 753 structures of intermediates before and after the enantioselective transition state of benzaldehyde propargylation, with activation energies (Δ*E*^‡^) computed at the B97D/TZV(2p,2d) level. SMILES strings (“fragment-based” SMILES) and “True” atom-maps are not provided with the original data set, these are taken from ref (38).

RXNMapper(107)-mapped versions of GDB7-22-TS and Cyclo-23-TS were obtained with the python package rxnmapper (version 0.3.0), using the default settings. The Proparg-21-TS set cannot be mapped, because the underlying libraries cannot process its SMILES string.^38^ Since RXNMapper sorts molecules in case of multiple reactants and/or products, which would complicate SMILES–xyz matching (see Section 5.3 below), we used a locally modified version that does not change the molecule order (the patch file is provided in the project repository at https://github.com/lcmd-epfl/EquiReact/tree/9d78892fe/data-curation/rxnmapper).

### 5.2 Data Splits

For each data set and splitting type, identical data splits were used for all the models compared. In each case, ten different splits are constructed with different random seeds.

Three different types of extrapolation split were used: scaffold-, molecular size- and property-based. Scaffold splitting^136,137^ clusters molecules based on their 2D backbones (such as Bemis–Murcko scaffolds^127^) and ensures that the clusters (scaffolds) belonging to the training, validation, and test sets do not overlap. Size-based splitting organizes the splits such that the reactions of the smallest molecules are in the training set and the reactions of the largest molecules are in validation and test. With property-based splits, one trains on reactions with higher barriers and predicts on reactions with lower barriers. This choice of splits reflects the relevant out-of-sample cases: larger molecules are more expensive to compute, and reactions with smaller barriers are desirable. Size- and property-based splits can also be organized in reverse order, where larger molecules are in the train set and smaller in test, or reactions with lower barrier in train and higher barrier in test.

For molecular size- and scaffold-based splits, the initial data shuffling affects the composition of the data sets. The nonzero standard deviations for property-based splits with neural networks arise from different organization of the data points into batches.

### 5.3 Matching SMILES Strings to xyz Geometries

3DReact makes use of both the graph structure of a molecule (as provided in the SMILES string) and the three-dimensional structure (in the xyz). The atoms in the graph are associated with the atomic coordinates provided in the xyz file. Thanks to the way the GDB7-22-TS data set^116^ was generated, the atomic coordinates can be easily matched to SMILES which in turn allows to atom-map reactants to products. However, we also tested RXNMapper-mapped SMILES which do not respect the same constraints. Therefore, for consistency, we use a SMILES–xyz matching procedure detailed below.

We construct molecular graphs from xyz using covalent radii and matched them to RDKit(120) molecular graphs obtained from SMILES with a labeled graph matching algorithm as implemented in NetworkX.^134,135^ This procedure is however unaware of chirality and double-bond stereochemistry, thus some of the matches might be incorrect. Still, it provides a flexible method that can be applied to any data set consisting of SMILES strings and xyz files.

The same procedure was applied to the Cyclo-23-TS data set in the few cases when the canonical SMILES have a different atom ordering than xyz.

### 5.4 xTB Geometry Generation

For the GDB7-22-TS and Cyclo-23-TS data sets, the starting structures were generated from SMILES using the distance-geometry embedding implemented in RDKit(120) with the srETKDGv3 settings.^138^ Ten conformations were produced per molecule, which were then energy-ranked with the MMFF94 implementation^139^ in RDKit, defaulting to UFF in case of missing parameters. The lowest energy conformer was retained. For the Proparg-21-TS set, the original B97D/TZV(2p,2d) geometries were used as a starting point, because the stereochemical and conformational diversity of this set cannot be completely encoded with SMILES. Therefore, MMFF94 will fail to generate an initial geometry from SMILES.

For all the sets, the starting structures were optimized at the GFN2-xTB semiempirical level of theory^129^ at the “loose” convergence level for a maximum of 1000 iterations using xTB(140) version 6.2 RC2. For 969 reactions of the GDB7-22-TS set and 491 reactions of the Cyclo-23-TS set, at least one of the participating molecules either could not converge to any reasonable configuration or converged to a structure not matching the SMILES. These reactions were excluded from the geometry quality tests (Section 3.4).

### 5.5 Model Training

3DReact was trained using the Adam optimizer^141^ with initial learning rate and weight decay parameters as hyperparameters. The learning rate was reduced by 40% after 60 epochs of no improvement in the validation MAE, as in ref (121). Models were trained for max. 512 epochs, using early stopping after 150 epochs of no improvement. The model with the best validation score was then used to make predictions on the test set.

The optimal model hyperparameters were searched within the following values: learning rate ∈ [5 × 10^–5^, 10^–4^, 5 × 10^–4^, 10^–3^]; weight decay parameter ∈ [10^–5^, 10^–4^, 10^–3^, 0]; node and edge features embedding size *n*_s_ ∈ [16, 32, 48, 64];  = 1 hidden space size *n*_v_ ∈ [16, 32, 48, 64]; number of edge features *n*_g_ ∈ [16, 32, 48, 64]; number of convolutional layers *n*_conv_ ∈ [2, 3]; radial cutoff *r*_max_ ∈ [2.5, 5.0, 10.0]; maximum number of atom neighbors *n*_neigh_ ∈ [10, 25, 50]; dropout probability *p*_d_ ∈ [0.0, 0.05, 0.1]; sum_mode ∈ [node, both]; combine_mode ∈ [mlp, diff, mean, sum]; graph_mode ∈ [energy, vector].

The hyperparameter search was done for the equivariant model EquiReact_S_ (without attention or mapping) using Bayesian search as implemented in Weights and Biases.^142^ Hydrogen atoms were excluded from the graphs. Sweeps were run for 128 epochs for the GDB7-22-TS and Proparg-21-TS sets, and for 256 epochs for the Cyclo-23-TS set on the first random split. The parameters resulting in the best validation error, summarized in Table S1, were used for all the other model settings.

### 5.6 Baseline Models

The ChemProp model^103^ is based on a CGR built from atom-mapped SMILES strings of reactants and products, which is then passed through the directed message-passing neural network chemprop(71,103,137) (version 1.5.0). The hyperparameters are taken from ref (38).

Molecular SLATM vectors were generated using the qml python package^143^ before being combined to form the reaction version SLATM_d_. SLATM_d_ is used with kernel ridge regression (KRR) models. The kernel functions and widths, and regularization parameters, were optimized on the first of the ten random splits, in line with how the hyperparameters were optimized for 3DReact. Unlike 3DReact, the hyperparameters for DFT and xTB geometries were optimized separately.

## Data Availability

The code is available as a GitHub repository at https://github.com/lcmd-epfl/EquiReact. The versions of the data sets used, as well as any processing applied to them, can be found in the same repository. The unprocessed results are available in the same same repository as well as at https://wandb.ai/equireact.

## Supporting Information Available

Detailing the architecture of the molecular channels; the 3DReact hyperparameters; the RMSE analogue of Table; the discussion of the model with a cross-attention surrogate for atom-mapping; extrapolation studies; some illustrative correlation plots for the GDB7-22-TS set; the model performance with and without explicit hydrogen atoms; and the geometry sensitivity analysis for the Cyclo-23-TS set (PDF)
