# Supplementary material for: 3DReact: Geometric Deep Learning for Chemical Reactions
Source: J Chem Inf Model. 2024 Jul 15;64(15):5771–85. doi: 10.1021/acs.jcim.4c00104 (PMC11323278; doi:10.1021/acs.jcim.4c00104)
Supplement: Supplementary file 1 — ci4c00104_si_001.pdf [file ci4c00104_si_001.pdf]

Supplementary Information

3DReact: Geometric deep learning for chemical reactions

Puck van Gerwen,[1,2] Ksenia R. Briling,[2,3] Charlotte Bunne,[2,3] Vignesh Ram Somnath,[2,3] Ruben Laplaza,[1,2] Andreas Krause,[2,3] and Clemence Corminboeuf[1,2,a)]

[1)] *Laboratory for Computational Molecular Design, Institute of Chemical Sciences and Engineering, École Polytechnique Fédérale de Lausanne, 1015 Lausanne, Switzerland*

[2)] *National Center for Competence in Research – Catalysis (NCCR-Catalysis), École Polytechnique Fédérale de Lausanne, 1015 Lausanne, Switzerland*

[3)] *Learning & Adaptive Systems Group, Department of Computer Science, ETH Zurich, 8092 Zurich, Switzerland*

(Dated: 25 June 2024)

CONTENTS

S1. Molecular channels S2

S2. Model hyperparameters S4

S3. Root-mean-square errors S5

S4. Cross-attention as a surrogate for atom-mapping in the "None" regime S6

S5. Extrapolation studies S7

S6. Correlation plots for the GDB7-22-TS dataset S9

S7. Performance with and without explicit hydrogen atoms S9

S8. Geometry sensitivity for the Cyclo-23-TS dataset S12

References S13

[a)] Electronic mail: clemence.corminboeuf@epfl.ch

S1. MOLECULAR CHANNELS

As briefly described in the main text, a molecule is represented as a distance-based graph where nodes describe atoms and edges describe bonds. Instead of explicitly using connectivity information, the "bonds" of atom a are formed with all the neighboring Neigh(a) atoms within the cutoff $r_{\max}$, all the (directed) bonds $\{(a, b)\}$ in the molecule forming set $\mathfrak{B}$. Initial node (atom) features $\{\mathbf{x}_a^{(0)}\}$ encode several cheminformatic features from `RDKit`,[S1] including atomic number, chirality tag (unspecified, tetrahedral, or other, including octahedral, square planar, allene-type, *etc.*), number of directly-bonded neighbors, number of rings, implicit valence, formal charge, number of attached hydrogens, number of radical electrons, hybridization, aromaticity, presence in rings of specified sizes from 3 to 7.

Inspired from related models,[S2] initial scalar edge (bond) features $\{\mathbf{e}_{ab}^{(0)}\}$ are projections of the atom distances $|\mathbf{r}_{ab}|$ onto n_g Gaussians uniformly spanning the line segment from 0 to $r_{\max}$ with the step $\Delta\mu = r_{\max}/(n_g - 1)$,

$$\mathbf{e}_{ab}^{(0)} = \mathbf{f}_1(|\mathbf{r}_{ab}|) \qquad \forall (a, b) \in \mathfrak{B}, \tag{S1}$$

$$\mathbf{f}_1(r) = \left\{ \exp\left(-\frac{1}{2} \left(\frac{r - n\Delta\mu}{\Delta\mu} \right)^2 \right) \right\} \quad n \in 0, \ldots, n_g{-}1. \tag{S2}$$

The tensorial edge features $\{\mathbf{z}_{ab}\}$, later used as filters, are projections of normalized difference vectors between atomic positions $\mathbf{r}_{ab}/|\mathbf{r}_{ab}|$ onto spherical harmonics Y_m^ℓ of $0 \le \ell \le 2$,

$$\mathbf{z}_{ab} \equiv \mathbf{z}_{ab}^{0e} \oplus \mathbf{z}_{ab}^{1o} \oplus \mathbf{z}_{ab}^{2e} = \mathbf{f}_2(\mathbf{r}_{ab}/|\mathbf{r}_{ab}|) \qquad \forall (a, b) \in \mathfrak{B}, \tag{S3}$$

$$\mathbf{f}_2(\mathbf{r}) = Y_0^0(\mathbf{r}) \oplus \left\{ Y_m^1(\mathbf{r}) \right\}_{|m| \le 1} \oplus \left\{ Y_m^2(\mathbf{r}) \right\}_{|m| \le 2}, \tag{S4}$$

where $\oplus$ denotes the concatenation operator, and the components of $\mathbf{z}_{ab}$ are labelled in superscript by the corresponding irreducible representation (ℓe for even parity and ℓo for odd parity) of the O(3) group.[S3] The initial $\mathbf{x}^{(0)}$ and $\mathbf{e}^{(0)}$ are then passed through embeddings to give $\mathbf{x}_a^{(1)}$ $\forall a$ and $\mathbf{e}_{ab}$ $\forall (a, b) \in \mathfrak{B}$.

The atomic representations $\{\mathbf{x}_a^{(1)}\}$ are updated by $n_{\text{conv}} \in \{2, 3\}$ equivariant convolutional layers:

$$\textbf{Layer 1} \begin{cases} \mathbf{w}_{ab}^{(1)} = \mathbf{g}_{31}(\mathbf{e}_{ab} \oplus \mathbf{x}_a^{(1)} \oplus \mathbf{x}_b^{(1)}) \quad \forall (a, b) \in \mathfrak{B} & \text{(S5)} \\ \mathbf{s}_b^{(1)} \equiv \mathbf{s}_b^{0e(1)} \oplus \mathbf{s}_b^{1o(1)} & \\ \quad = \frac{1}{\text{Neigh}(b)} \sum_{a:(a,b) \in \mathfrak{B}} \mathbf{t}_1(\mathbf{x}_a^{(1)}, \mathbf{z}_{ab}, \mathbf{w}_{ab}^{(1)}) \quad \forall b & \text{(S6)} \\ \mathbf{x}^{0e(2)} = \mathbf{x}^{(1)} + \mathbf{s}^{0e(1)} & \text{(S7)} \\ \mathbf{x}^{(2)} = \mathbf{x}^{0e(2)} \oplus \mathbf{s}^{1o(1)} & \text{(S8)} \end{cases}$$

$$\textbf{Layer 2} \begin{cases} \mathbf{w}_{ab}^{(2)} = \mathbf{g}_{32}(\mathbf{e}_{ab} \oplus \mathbf{x}_a^{0e(2)} \oplus \mathbf{x}_b^{0e(2)}) \quad \forall (a, b) \in \mathfrak{B} & \text{(S9)} \\ \mathbf{s}_b^{(2)} \equiv \mathbf{s}_b^{0e(2)} \oplus \mathbf{s}_b^{1o(2)} \oplus \mathbf{s}_b^{1e(2)} & \\ \quad = \frac{1}{\text{Neigh}(b)} \sum_{a:(a,b) \in \mathfrak{B}} \mathbf{t}_2(\mathbf{x}_a^{(2)}, \mathbf{z}_{ab}, \mathbf{w}_{ab}^{(2)}) \quad \forall b & \text{(S10)} \\ \mathbf{x}^{0e(3)} = \mathbf{x}^{0e(2)} + \mathbf{s}^{0e(2)} & \text{(S11)} \\ \mathbf{x}^{(3)} = \mathbf{x}^{0e(3)} \oplus \left(\mathbf{s}^{1o(1)} + \mathbf{s}^{1o(2)} \right) \oplus \mathbf{s}^{1e(2)} & \text{(S12)} \end{cases}$$

$$\textbf{Layer 3} \begin{cases} \mathbf{w}_{ab}^{(3)} = \mathbf{g}_{33}(\mathbf{e}_{ab} \oplus \mathbf{x}_a^{0e(3)} \oplus \mathbf{x}_b^{0e(3)}) \quad \forall (a, b) \in \mathfrak{B} & \text{(S13)} \\ \mathbf{s}_b^{(3)} \equiv \mathbf{s}_b^{0e(3)} \oplus \mathbf{s}_b^{1o(3)} \oplus \mathbf{s}_b^{1e(3)} \oplus \mathbf{s}_b^{0o(3)} & \\ \quad = \frac{1}{\text{Neigh}(b)} \sum_{a:(a,b) \in \mathfrak{B}} \mathbf{t}_3(\mathbf{x}_a^{(3)}, \mathbf{z}_{ab}, \mathbf{w}_{ab}^{(3)}) \quad \forall b & \text{(S14)} \\ \mathbf{x}^{\text{out}} = \left(\mathbf{x}^{0e(3)} + \mathbf{s}^{0e(3)} \right) \oplus \mathbf{s}^{0o(3)}. & \text{(S15)} \end{cases}$$

In **Layer 1**, for example, $\mathbf{s}_b^{(1)} \equiv \mathbf{s}_b^{0e(1)} \oplus \mathbf{s}_b^{1o(1)}$ means that the result of the function $\mathbf{t}_1$ consists of scalars ($0e$) and vectors ($1o$) that can be treated separately. Each function $\mathbf{t}_n(\mathbf{x}, \mathbf{z}, \mathbf{w})$ is a fully-connected weighted tensor product,

as defined in `e3nn`,[S4] in the form of

$$\mathbf{t}_n(\mathbf{x},\mathbf{z},\mathbf{w}) = \bigoplus_k \mathbf{t}_k^{(n)}, \quad \mathbf{t}_k^{(n)} = \sum_{uv} w_{uvk}^{(n)} \mathbf{x}_u \otimes \mathbf{z}_v, \tag{S16}$$

where $\{k,u,v\}$ index individual tensors. Note that a tensor here refers to the mathematical object that obeys certain transformation laws, not the multi-dimensional array. The functions $\{\mathbf{t}_n\}$ are specified by signatures of irreducible representations (irreps) of two input and one output O(3) tensors. The output tensor is a combination of weighted sums of paths (pairs of input irreps) leading to each output irrep. The irreducible representation (irrep) sequence in each layer from 1–3 is illustrated in Figure S1,

For example, irreps of $\mathbf{x}_a^{(0)}$ and $\mathbf{z}_{ab}$ are $(n_s{\times}0e)$ and $(0e \oplus 1o \oplus 2e)$, respectively, because the former consists of n_s scalars and the latter is a direct sum of projections onto spherical harmonics of $\ell = 0, 1, 2$. The desired output irreps are chosen deliberately from the possible products of the input irreps and can have any shape, so the signature of function $\mathbf{t}_1$ is

$$\mathbf{t}_1 : (n_s{\times}0e) \otimes (0e \oplus 1o \oplus 2e) \to (n_s{\times}0e \oplus n_v{\times}1o). \tag{S17}$$

Thus two paths are created,

$$(n_s{\times}0e) \otimes (1{\times}0e) \to (n_s{\times}0e) \quad \text{with } n_s{\times}1{\times}n_s \text{ weights,} \tag{S18}$$

$$(n_s{\times}0e) \otimes (1{\times}1o) \to (n_v{\times}1o) \quad \text{with } n_s{\times}1{\times}n_v \text{ weights.} \tag{S19}$$

The output contains n_s scalars and n_v vectors. There is one tensor per each bond (a,b), so a tensor $\mathbf{s}_b^{(1)}$ for atom b is an average of $\mathbf{t}_1(\mathbf{x}_a^{(1)}, \mathbf{z}_{ab}, \mathbf{w}_{ab}^{(1)})$ over its neighbors $\{a\}$. It is used to update $\mathbf{x}$ which is convoluted with $\mathbf{z}_a$ two more times using functions with signatures

$$\mathbf{t}_2 : (n_s{\times}0e \oplus n_v{\times}1o) \otimes (0e \oplus 1o \oplus 2e) \to (n_s{\times}0e \oplus n_v{\times}1o \oplus n_v{\times}1e), \tag{S20}$$

adding n_v pseudovectors ($n_s^2 + 2n_sn_v + 3n_v^2$ weights total), and

$$\mathbf{t}_3 : (n_s{\times}0e \oplus n_v{\times}1o \oplus n_v{\times}1e) \otimes (0e \oplus 1o \oplus 2e) \to (n_s{\times}0e \oplus n_v{\times}1o \oplus n_v{\times}1e \oplus n_s{\times}0o), \tag{S21}$$

adding n_s pseudoscalars ($n_s^2 + 3n_sn_v + 6n_v^2$ weights total).

To obtain the weights $\mathbf{w}^{(n)}$ for each convolutional layer n, the spherical parts of $\mathbf{x}_a^{(n)}$ and $\mathbf{x}_b^{(n)}$ are concatenated with the bond features $\mathbf{e}_{ab}$ and passed through a multi-layer perceptron $\mathbf{g}_{3n}$.

The output of the equivariant molecular channels is the local molecular representation $\mathbf{X} \in \mathbb{R}^{N_\text{at} \times D}$ corresponding to N_at atoms associated with D features. Depending on the `sum_mode` hyperparameter, it is constructed either from the node features $\{\mathbf{x}_a^\text{out}\}$ (`node` mode) or both node and edge features $\{\mathbf{x}_a^\text{out} \oplus \sum_{b:(a,b)\in\mathfrak{B}} \mathbf{e}_{ab}^{(0)}\}$ (`both` mode). In the case of $n_\text{conv} = 2$, the vectors $\{\mathbf{x}_a^{0e(3)}\}$ are taken to construct the molecular representation.

Note that even through the resulting node features $\mathbf{X}$ should be invariant (with $n_\text{conv} = 2$) or equivariant (with $n_\text{conv} = 3$) with coordinate inversion, the chirality tag, if present in the SMILES, changes the initial node features $\{\mathbf{x}_a^{(0)}\}$ and may lead to different representations for two enantiomers.

Above is described the architecture of EquiReact, the equivariant option of 3DReact. The invariant option (InReact) is implemented as a simplified version of the latter: the spherical harmonics filters take only $\ell = 0$ (hence $\mathbf{z}_{ab} = 1\ \forall(a,b) \in \mathfrak{B}$), the convolutional layers signatures become

$$\mathbf{t}'_n : (n_s{\times}0e) \otimes (0e) \to (n_s{\times}0e), \quad n \in \{1,2,3\}, \tag{S22}$$

and Eq. S16 is simplified to a dot product

$$\mathbf{t}'_n(\mathbf{x},\mathbf{z},\mathbf{w}) \equiv \mathbf{t}'_n(\mathbf{x},\mathbf{w}) = \bigoplus_k \mathbf{t}'^{(n)}_k, \quad \mathbf{t}_k^{(n)} = \sum_u w_{uk}^{(n)} x_u. \tag{S23}$$

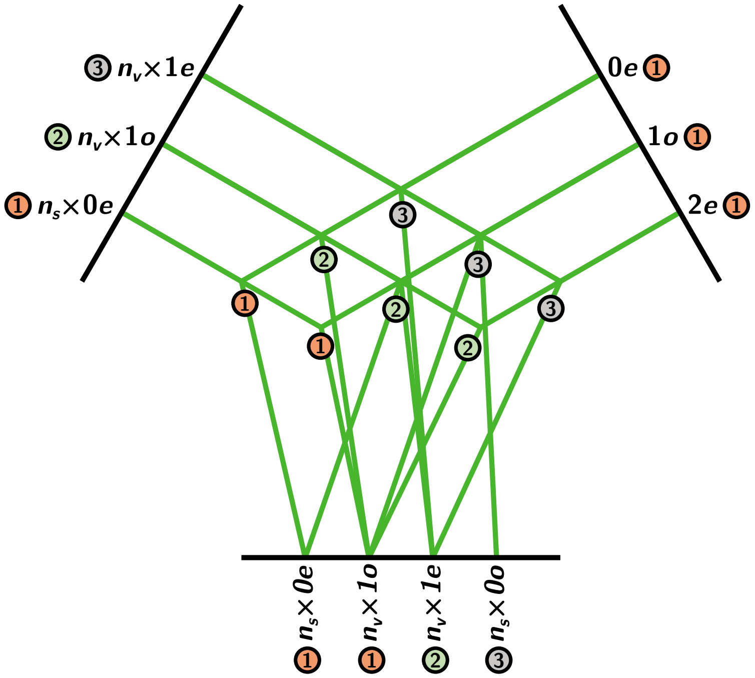

FIG. S1. Irrep sequence in the (1), (2), (3) convolutional layers of EquiReact. Input irreps are on the left (hidden atom and bond features) and right (spherical harmonics filters), output irreps are at the bottom, and paths that connect them are in the middle in green. Note that formally $2e$ is present in the right input already at the first layer, but does not contribute to the output.

S2. MODEL HYPERPARAMETERS

The best model hyperparameters obtained after the sweep on EquiReact are summarized in Table S1. These are the hyperparameters used in all 3DReact models in the main text.

Parameter	GDB7-22-TS	Cyclo-23-TS	Proparg-21-TS
n_s	64	64	64
n_v	64	48	64
n_g	32	48	64
n_{conv}	2	2	3
$r_{\max}$, Å	2.5	2.5	5
n_{neigh}	10	50	10
p_d	0.05	0.1	0.05
`sum_mode`	`both`	`node`	`node`
`combine_mode`	`diff`	`diff`	`diff`
`graph_mode`	`vector`	`energy`	`vector`
learning rate	$5 \cdot 10^{-4}$	10^{-3}	10^{-3}
weight decay	10^{-5}	10^{-5}	10^{-5}

TABLE S1. Best model hyperparameters as a result of the sweeps.

S3. ROOT-MEAN-SQUARE ERRORS

The results presented in Table 2 and Table 1 are repeated in Table S2 using root mean squared errors (RMSEs) rather than mean absolute errors (MAEs) as the performance metric.

Dataset (property, units)	Atom-mapping regime	ChemProp	SLATM$_d$+KRR	InReact	EquiReact
Random splits					
GDB7-22-TS ($\Delta E^\ddagger$, kcal/mol)	True	7.6 ± 0.3	—	8.4 ± 0.4	8.4 ± 0.3
	RXNMapper	9.57 ± 0.28	—	9.9 ± 0.4	10.0 ± 0.4
	None	13.2 ± 0.5	10.8 ± 0.4	10.5 ± 0.4	10.4 ± 0.5
Cyclo-23-TS ($\Delta G^\ddagger$, kcal/mol)	True	3.70 ± 0.20	—	3.33 ± 0.18	3.24 ± 0.21
	RXNMapper	3.72 ± 0.16	—	3.31 ± 0.21	3.29 ± 0.26
	None	3.73 ± 0.23	3.65 ± 0.22	3.34 ± 0.18	3.24 ± 0.23
Proparg-21-TS ($\Delta E^\ddagger$, kcal/mol)	True	1.97 ± 0.16	—	0.58 ± 0.16	0.52 ± 0.14
	None	2.01 ± 0.19	0.52 ± 0.09	0.60 ± 0.16	0.49 ± 0.09
Scaffold splits					
GDB7-22-TS ($\Delta E^\ddagger$, kcal/mol)	True	10.6 ± 0.9	—	11.6 ± 1.0	11.5 ± 1.2
	RXNMapper	12.8 ± 0.9	—	13.3 ± 1.0	13.3 ± 1.1
	None	17.4 ± 0.8	15.2 ± 1.3	14.5 ± 1.1	14.4 ± 1.1
Cyclo-23-TS ($\Delta G^\ddagger$, kcal/mol)	True	3.9 ± 0.4	—	3.7 ± 0.4	3.7 ± 0.4
	RXNMapper	3.9 ± 0.4	—	3.7 ± 0.4	3.7 ± 0.5
	None	4.1 ± 0.4	4.0 ± 0.4	3.7 ± 0.4	3.7 ± 0.4
Proparg-21-TS ($\Delta E^\ddagger$, kcal/mol)	True	2.2 ± 0.3	—	0.71 ± 0.22	0.65 ± 0.17
	None	2.2 ± 0.4	0.62 ± 0.16	0.68 ± 0.18	0.64 ± 0.19

TABLE S2. Performance as measured in root-mean-square errors (RMSEs) of predictions of 3DReact (InReact and EquiReact) *vs.* ChemProp and SLATM$_d$. 3DReact$_M$ is used for the "True" and "RXNMapper" regimes, and 3DReact$_S$ is used for the "None" regime. RMSEs are averaged over 10 folds of random/scaffold 80/10/10 splits (training/validation/test) and reported together with standard deviations across folds.

S4. CROSS-ATTENTION AS A SURROGATE FOR ATOM-MAPPING IN THE "NONE" REGIME

Since the "True" atom-mapping regime allowed for a significantly improved model over the "RXNMapper" and "None" regimes for the GDB7-22-TS dataset, we thought that a model based on cross-attention between reactants and products (3DReact$_X$) could provide a competitive surrogate to the atom-mapping-based models.

Given queries $\mathbf{Q} \in \mathbb{R}^{N\times D}$, keys $\mathbf{K} \in \mathbb{R}^{M\times D}$ and values $\mathbf{V} \in \mathbb{R}^{M\times D}$, attention is computed as

$$\mathbf{A} = \mathrm{softmax}\left(\frac{\mathbf{QK}^T}{\sqrt{D}}\right) \tag{S24}$$

and the "reordered" values $\mathbf{Y}$ are

$$\mathbf{Y} = \mathbf{AV}. \tag{S25}$$

We used the implementation of this scaled-dot-product attention[S5] in `PyTorch`'s[S6] `MultiheadAttention` (`PyTorch` version 1.12.1). The representations are re-ordered using Eq. S24 and Eq. S25 with $\mathbf{Q}$ as the vector representation of reactants, $\mathbf{K}$ and $\mathbf{V}$ as the vector representations of products, and vice versa (thus here $N = M = N_{\mathrm{at}}$).

However, we found that the cross-attention module does not improve over the simple model 3DReact$_S$, based only on geometries of isolated reactants and products without any information exchange between them, for all three datasets (Table S3).

Dataset (property, units)	InReact$_X$	InReact$_S$	EquiReact$_X$	EquiReact$_S$
GDB7-22-TS ($\Delta E^\ddagger$, kcal/mol)	6.68 ± 0.27	6.56 ± 0.26	6.7 ± 0.3	6.53 ± 0.28
Cyclo-23-TS ($\Delta G^\ddagger$, kcal/mol)	2.53 ± 0.09	2.39 ± 0.05	2.43 ± 0.09	2.31 ± 0.09
Proparg-21-TS ($\Delta E^\ddagger$, kcal/mol)	0.34 ± 0.05	0.34 ± 0.06	0.33 ± 0.06	0.31 ± 0.06

TABLE S3. Performance of the alternative models 3DReact$_X$ and 3DReact$_S$ in the None mapping mode.

Investigating further for the GDB7-22-TS dataset, we wanted to find out whether the attention module could infer atom-mapping from an easier, supervised learning scenario. The Mapper model took the graphs of reactants and products as input, as for 3DReact. Since the atom indices in the reactants were ordered sequentially $(1, 2, 3, \dots)$, the objective was to learn the permutation of the atom indices in the products, in order to map them to the reactants correctly. The true atom maps provided by the GDB7-22-TS set[S7] were used to train and validate the model. The permutation was learned using cross-attention between atoms in reactants and products (Eq. S24) using queries $\mathbf{Q}$ from the vector representation of reactants and keys $\mathbf{K}$ from vector representations of products.

The model was trained using the Adam optimizer with default parameters. The hyperparameters used for the 3DReact (EquiReact) components of the Mapper model (to construct the molecular representations) are summarized in Table S4.

Parameter	n_s	n_v	n_g	n_{conv}	$r_{\max}$	n_{neigh}	p_d
Value	16	16	32	2	10	20	0.1

TABLE S4. Hyperparameters used to construct molecular components in the Mapper model.

The optimization objective was to minimize the cross-entropy loss between the true mapping permutation and the permutation learned by the model. Mapper was run for 100 epochs on a single random 80/10/10 split of the GDB7-22-TS dataset. The resulting training and validation curves are shown in Figure S2.

While the model learns, both the training and validation accuracy saturate at around 70%, suggesting there is an upper bound to the accuracy of the Mapper model. This may be related to the unconstrained nature of the optimization: while the predicted maps can converge to any integer value, each integer prediction should be taken from the pool of integers (atom-map labels) in the reactants, and appear only once. The architecture is also much simpler than that of RXNMapper[S8] for example.

Nevertheless, the fact that the model is able to learn atom-mapping to a reasonable degree of accuracy suggests that the cross-attention module in 3DReact could mimic a low-quality atom-mapping. Therefore, 3DReact$_X$ likely fails to improve model performance because the unsupervised atom-mapping task is embedded in a broader learning context. It seems that unlike the supervised task of reaction outcome prediction,[S8] a graph-based model trained to predict reaction barriers does not learn chemical signatures like atom-mapping as part of the training process. Other information (like that based on the geometries of reactants and products) is instead exploited to make good predictions.

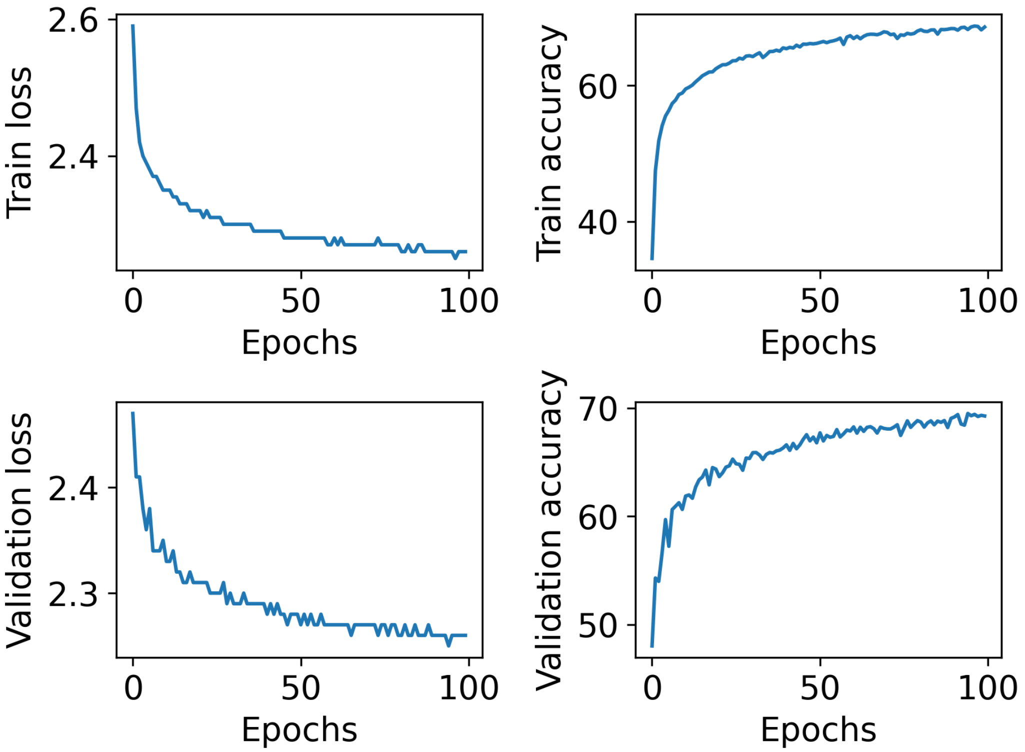

FIG. S2. Evolution of the training loss, training accuracy (%), validation loss, validation accuracy (%) for the Mapper model, trained to learn atom-mapping in a supervised setting.

S5. EXTRAPOLATION STUDIES

Table S2 shows the results for an extensive set of extrapolation studies, both for InReact and EquiReact as well as for the baseline models. Property-based splits order the training/validation/test sets according to the target value in ascending or descending order. The nonzero standard deviations for neural network models arise from different organization of the datapoints into batches. Size-based splits instead order the datapoints into training/validation/test sets according to molecular size, *i.e.*, number of heavy atoms (reactant size for GDB7-22-TS and Proparg-21-TS, product size for Cyclo-23-TS), again sorted in ascending or descending order. Since the molecular size is a discrete variable, the initial data shuffling affects the composition of the sets and leads to the non-zero standard deviations for all the models.

In the size-based splits, ascending is more challenging than descending, since a model trained on large molecules also has a notion of atoms in smaller molecules. In principle, property-based splits are equally challenging as ascending or descending, but due to asymmetry in the data distributions, ascending seems to result in higher test MAEs for all models.

Dataset (property, units)	Atom-mapping regime	ChemProp	SLATM$_d$+KRR	InReact	EquiReact
Scaffold splits					
GDB7-22-TS ($\Delta E^\ddagger$, kcal/mol)	True	7.1 ± 0.6	—	7.8 ± 0.7	7.8 ± 0.8
	RXNMapper	8.7 ± 0.8	—	9.2 ± 0.8	9.1 ± 0.8
	None	12.9 ± 0.6	$\mathbf{11.0 \pm 1.0}$	$\mathbf{10.1 \pm 0.9}$	$\mathbf{10.0 \pm 0.9}$
Cyclo-23-TS ($\Delta G^\ddagger$, kcal/mol)	True	2.92 ± 0.24	—	2.79 ± 0.18	2.72 ± 0.18
	RXNMapper	2.92 ± 0.23	—	2.77 ± 0.22	2.71 ± 0.23
	None	3.1 ± 0.3	2.97 ± 0.22	2.76 ± 0.22	2.72 ± 0.19
Proparg-21-TS ($\Delta E^\ddagger$, kcal/mol)	True	1.64 ± 0.21	—	$\mathbf{0.44 \pm 0.11}$	$\mathbf{0.40 \pm 0.08}$
	None	1.64 ± 0.19	$\mathbf{0.36 \pm 0.05}$	$\mathbf{0.45 \pm 0.10}$	$\mathbf{0.41 \pm 0.09}$
Property-based splits (ascending)					
GDB7-22-TS ($\Delta E^\ddagger$, kcal/mol)	True	$\mathbf{29.0 \pm 0.9}$	—	30.9 ± 0.6	30.9 ± 0.5
	RXNMapper	31.7 ± 0.9	—	33.0 ± 0.4	32.2 ± 0.6
	None	36.0 ± 0.4	**34.80**	35.3 ± 0.3	35.3 ± 0.4
Cyclo-23-TS ($\Delta G^\ddagger$, kcal/mol)	True	11.7 ± 0.3	—	$\mathbf{9.0 \pm 0.6}$	$\mathbf{9.3 \pm 0.4}$
	RXNMapper	12.1 ± 0.3	—	$\mathbf{8.8 \pm 0.7}$	$\mathbf{8.8 \pm 0.6}$
	None	11.9 ± 0.3	12.14	$\mathbf{9.5 \pm 0.3}$	$\mathbf{9.6 \pm 0.4}$
Proparg-21-TS ($\Delta E^\ddagger$, kcal/mol)	True	5.10 ± 0.12	—	$\mathbf{4.31 \pm 0.15}$	$\mathbf{4.08 \pm 0.11}$
	None	5.09 ± 0.13	5.83	$\mathbf{4.53 \pm 0.16}$	$\mathbf{4.33 \pm 0.14}$
Property-based splits (descending)					
GDB7-22-TS ($\Delta E^\ddagger$, kcal/mol)	True	24.0 ± 1.1	—	24.9 ± 0.9	25.0 ± 0.7
	RXNMapper	25.7 ± 0.7	—	26.8 ± 0.9	26.4 ± 0.4
	None	30.3 ± 0.5	31.08	30.6 ± 0.6	31.0 ± 0.3
Cyclo-23-TS ($\Delta G^\ddagger$, kcal/mol)	True	6.1 ± 0.4	—	$\mathbf{5.03 \pm 0.28}$	$\mathbf{4.8 \pm 0.5}$
	RXNMapper	5.9 ± 0.8	—	5.2 ± 0.4	4.6 ± 0.5
	None	6.22 ± 0.30	6.46	$\mathbf{5.18 \pm 0.17}$	$\mathbf{5.07 \pm 0.28}$
Proparg-21-TS ($\Delta E^\ddagger$, kcal/mol)	True	3.33 ± 0.20	—	$\mathbf{1.58 \pm 0.12}$	$\mathbf{1.69 \pm 0.20}$
	None	3.28 ± 0.23	2.02	$\mathbf{1.70 \pm 0.11}$	2.13 ± 0.27
Size-based splits (ascending)					
GDB7-22-TS ($\Delta E^\ddagger$, kcal/mol)	True	$\mathbf{5.62 \pm 0.20}$	—	6.41 ± 0.25	6.36 ± 0.24
	RXNMapper	$\mathbf{6.97 \pm 0.25}$	—	7.52 ± 0.19	7.55 ± 0.28
	None	10.39 ± 0.14	$\mathbf{8.06 \pm 0.16}$	$\mathbf{8.10 \pm 0.22}$	$\mathbf{8.11 \pm 0.23}$
Cyclo-23-TS ($\Delta G^\ddagger$, kcal/mol)	True	4.57 ± 0.15	—	$\mathbf{3.86 \pm 0.13}$	$\mathbf{3.86 \pm 0.11}$
	RXNMapper	4.68 ± 0.13	—	$\mathbf{3.91 \pm 0.13}$	$\mathbf{3.89 \pm 0.21}$
	None	4.57 ± 0.11	4.79 ± 0.03	$\mathbf{3.96 \pm 0.13}$	$\mathbf{3.96 \pm 0.16}$
Proparg-21-TS ($\Delta E^\ddagger$, kcal/mol)	True	1.537 ± 0.023	—	$\mathbf{0.52 \pm 0.07}$	$\mathbf{0.48 \pm 0.05}$
	None	1.538 ± 0.026	$\mathbf{0.504 \pm 0.021}$	0.63 ± 0.06	$\mathbf{0.55 \pm 0.05}$
Size-based splits (descending)					
GDB7-22-TS ($\Delta E^\ddagger$, kcal/mol)	True	$\mathbf{3.25 \pm 0.05}$	—	3.78 ± 0.14	3.74 ± 0.10
	RXNMapper	4.61 ± 0.15	—	4.75 ± 0.12	4.74 ± 0.13
	None	8.36 ± 0.22	$\mathbf{4.85 \pm 0.07}$	$\mathbf{4.90 \pm 0.11}$	$\mathbf{4.90 \pm 0.11}$
Cyclo-23-TS ($\Delta G^\ddagger$, kcal/mol)	True	2.85 ± 0.08	—	2.74 ± 0.10	2.72 ± 0.06
	RXNMapper	2.88 ± 0.06	—	2.77 ± 0.09	2.72 ± 0.07
	None	2.85 ± 0.07	2.80 ± 0.03	2.85 ± 0.07	2.76 ± 0.09
Proparg-21-TS ($\Delta E^\ddagger$, kcal/mol)	True	1.30 ± 0.04	—	$\mathbf{0.278 \pm 0.021}$	$\mathbf{0.263 \pm 0.023}$
	None	1.30 ± 0.04	0.307 ± 0.011	$\mathbf{0.236 \pm 0.029}$	$\mathbf{0.226 \pm 0.028}$

TABLE S5. Performance as measured in mean absolute errors (MAEs) of predictions of 3DReact (InReact and EquiReact) *vs.* ChemProp and SLATM$_d$. MAEs are averaged over 10 folds of 80/10/10 splits (training/validation/test) and reported together with standard deviations across folds. Lowest errors are highlighted in bold, if there are statistically meaningful differences between models in each regime/dataset/split type tested.

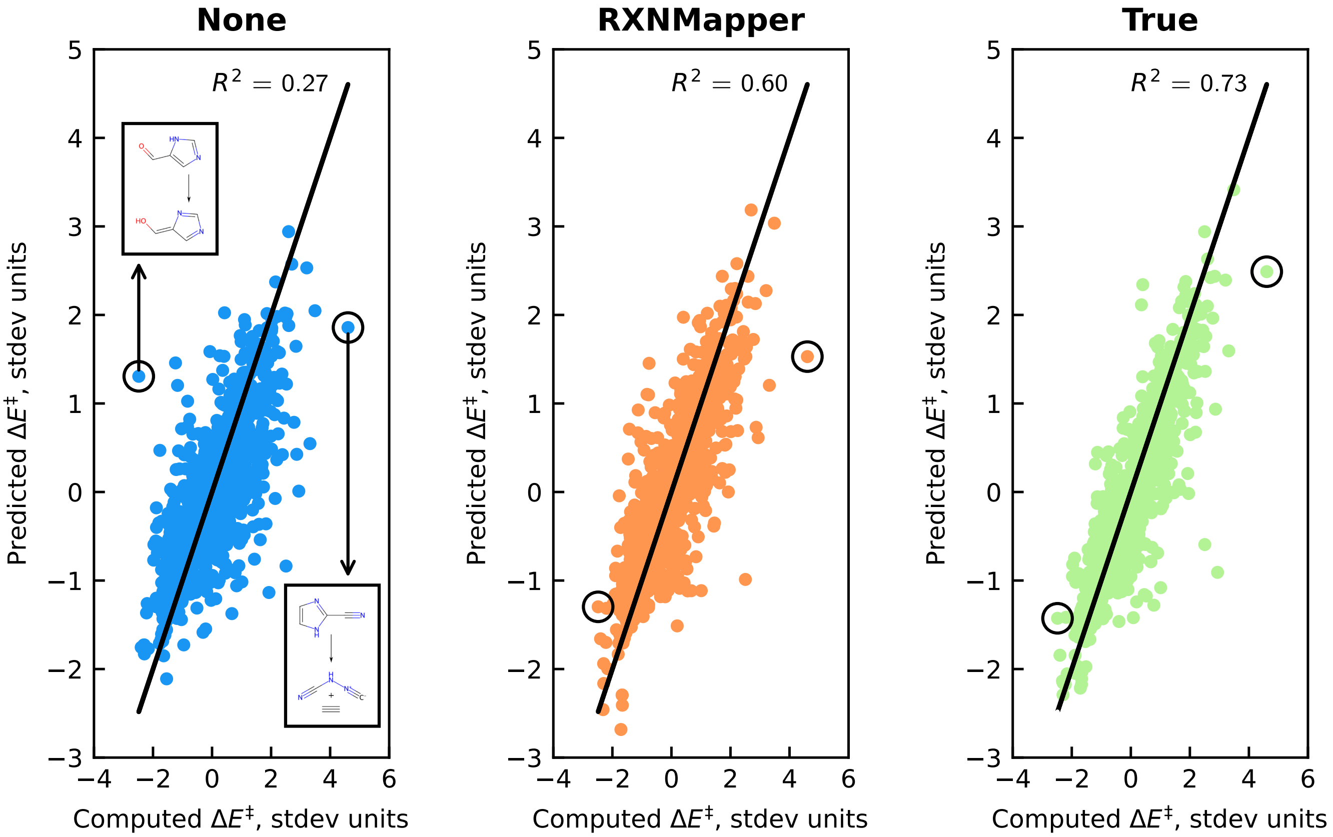

FIG. S3. Correlation plots of predicted with 3DReact (InReact) $\Delta E^{\ddagger}$ values *vs.* true (computed) labels for the first reactant-based scaffold split on the GDB7-22-TS dataset. The same test reactions are highlighted in each subplot.

S6. CORRELATION PLOTS FOR THE GDB7-22-TS DATASET

Figure S3 illustrates that for the same scaffold split, from "None" to "RXNMapper" to "True" the coefficient of determination (R^2) increases and outliers successively move closer to the $y = x$ line.

S7. PERFORMANCE WITH AND WITHOUT EXPLICIT HYDROGEN ATOMS

The results presented in the main text were for models built from molecular graphs constructed without explicit inclusion of hydrogen atoms as nodes. The performance for models including H-nodes is shown in Table S6.

For the GDB7-22-TS dataset, the best-performing model in the "True" regime (ChemProp) is with explicit Hs. Since this set includes H_2-abstraction reactions, the explicit inclusion of hydrogen in combination with the reaction mechanism in the form of atom-mapping is particularly informative. Interestingly, 3DReact "True" and "None" does not suffer when removing H atoms. In the "RXNMapper" regime, the inclusion of H-nodes increases the errors especially for ChemProp. The atom-mapping provided by RXNMapper is inferred as a consequence of a related task: predicting randomly masked parts of a reaction sequence (reaction SMILES).[S8] Since there are few H atoms in reaction SMILES strings (typically they are implicit), it is natural that RXNMapper struggles to map H atoms in reactions.

For the Cyclo-23-TS and Proparg-21-TS datasets, no models benefit from the inclusion of explicit Hs in any regime. Since larger molecules participate in these reactions which never consist purely of H_2-abstraction, the reactions are well-described without an explicit description of H atoms. The cost of the message passing increases considerably when including H-nodes, and 3DReact's performance is not strongly correlated with their inclusion. This coupled with the fact that atom-mapping tools usually do not map hydrogens, as well as the fact that most atom-mapped reaction SMILES have maps only for heavy atoms, resulted in the decision that we use the models without explicit

H-nodes in ChemProp and 3DReact.

Dataset (property, units)	H mode	Atom mapping regime					
		True		RXNMapper		None	
		ChemProp	3DReact$_M$	ChemProp	3DReact$_M$	ChemProp	3DReact$_S$
GDB7-22-TS	with	**4.12 ± 0.13**	4.90 ± 0.16	6.36 ± 0.09	6.24 ± 0.21	8.87 ± 0.28	**6.54 ± 0.25**
($\Delta E^{\ddagger}$, kcal/mol)	w/o	4.35 ± 0.15	4.93 ± 0.18	**5.69 ± 0.17**	6.03 ± 0.26	9.04 ± 0.21	**6.56 ± 0.26**
Cyclo-23-TS	with	—	**2.33 ± 0.07**	2.79 ± 0.12	**2.37 ± 0.07**	2.76 ± 0.10	**2.38 ± 0.08**
($\Delta G^{\ddagger}$, kcal/mol)	w/o	2.69 ± 0.10	**2.39 ± 0.08**	2.71 ± 0.07	**2.37 ± 0.07**	2.71 ± 0.12	**2.39 ± 0.05**
Proparg-21-TS	with	1.55 ± 0.16	**0.38 ± 0.07**	—	—	1.54 ± 0.14	**0.37 ± 0.05**
($\Delta E^{\ddagger}$, kcal/mol)	w/o	1.53 ± 0.14	**0.33 ± 0.07**	—	—	1.56 ± 0.16	**0.34 ± 0.06**

TABLE S6. Performance of 3DReact (InReact) with explicit hydrogens as nodes in the graphs (H mode "with") and without (H mode "w/o", as in the main text). Cyclo-23-TS "True" with Hs is missing because the dataset provides atom maps for heavy atoms only, while 3DReact does not suffer from that since it uses atom-mapped xyz files (see Section 5.1). MAEs are averaged over 10 folds of 80/10/10 splits (training/validation/test) and reported together with standard deviations across folds. Lowest errors, if statistically relevant, in each dataset/mapping regime are highlighted in bold.

Figure S4 illustrates the performance of 3DReact "True" trained with explicit H-nodes. Compared to the model trained without explicit H-nodes (Figure 7 in the main text), the error distribution in the different reaction types is more uniform, since the GDB7-22-TS dataset consists of many reactions involving breaking and forming H–X bonds, which are better captured using a model with explicit H atoms. Nevertheless, 3DReact without explicit Hs already performs well across the different reaction classes, in exchange for faster message passing.

Table S7 shows the performance of SLATM$_d$+KRR with SLATM features constructed in three ways: with Hs ("with"), with H features removed after building the representation ("w/o after") and H atoms removed before building the representation ("w/o before"). The first variation is the standard construction of SLATM features.[S9] The second variation is the closest possible version to "implicit" H regimes of ChemProp and 3DReact since the H-only features (H–H and X–H–Y bins) are removed. Nevertheless, other H-containing bins, *e.g.* C–H or C–C–H, are still incorporated. The last version removes H atoms completely from the system. For the GDB7-22-TS set, which is dependent on an accurate description of X–H bonds, errors increase systematically on the removal of H information. The same is seen to a lesser extent in the Cyclo-23-TS set. For the Proparg-21-TS, X–H bond changes have a minimal impact on the description of the reactions. SLATM$_d$ is run with explicit H atoms by default, since the representation is constructed from the xyz file directly without use of SMILES strings. Regardless of whether H atoms are in the SMILES strings, they are always present in the xyz file and therefore excluding H is nonsensical for SLATM.

H mode	GDB7-22-TS ($\Delta E^{\ddagger}$, kcal/mol)	Cyclo-23-TS ($\Delta G^{\ddagger}$, kcal/mol)	Proparg-21-TS ($\Delta E^{\ddagger}$, kcal/mol)
with	6.89 ± 0.20	2.65 ± 0.08	0.33 ± 0.04
w/o after	7.11 ± 0.20	2.69 ± 0.08	0.33 ± 0.04
w/o before	8.04 ± 0.22	2.82 ± 0.09	0.34 ± 0.04

TABLE S7. Performance of SLATM$_d$+KRR with hydrogens in the representation (H mode "with", as in the main text), with hydrogens excluded after computing the representation (H mode "w/o after"), and with hydrogens excluded before computing the representation (H mode "w/o before"). MAEs are averaged over 10 folds of 80/10/10 splits (training/validation/test) and reported together with standard deviations across folds.

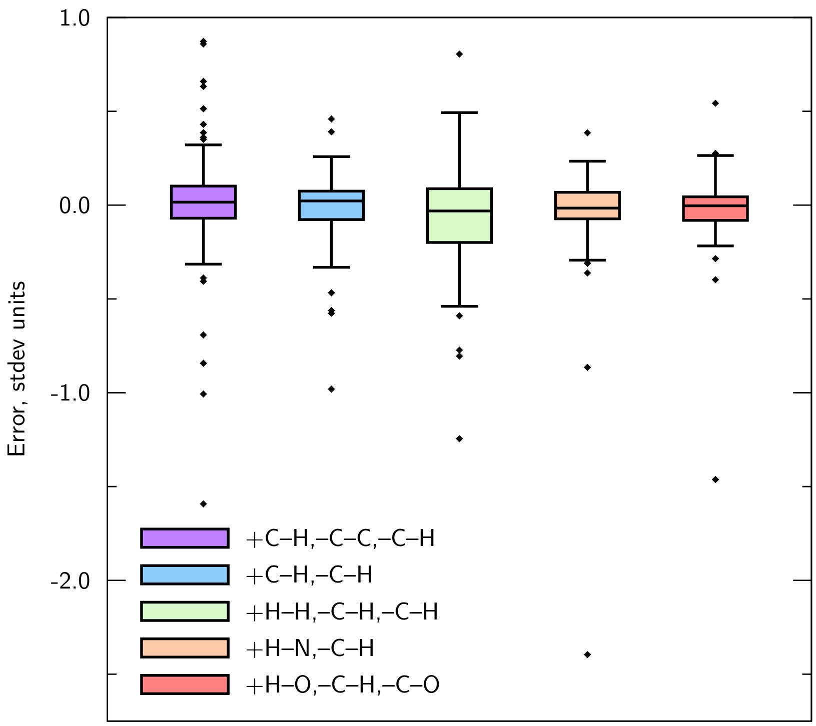

FIG. S4. Box plots illustrating how 3DReact (InReact$_M$ "True") performs for the most common reaction types in the GDB7-22-TS set, when using explicit H nodes in the graphs. The boxes range from the first to the third quartile of the datapoints. The whiskers limit 90% of the datapoints and the individual points illustrate outliers. The points correspond to the test set of the first random split. The errors are given in the target standard deviation (stdev) units (21.8 kcal/mol).

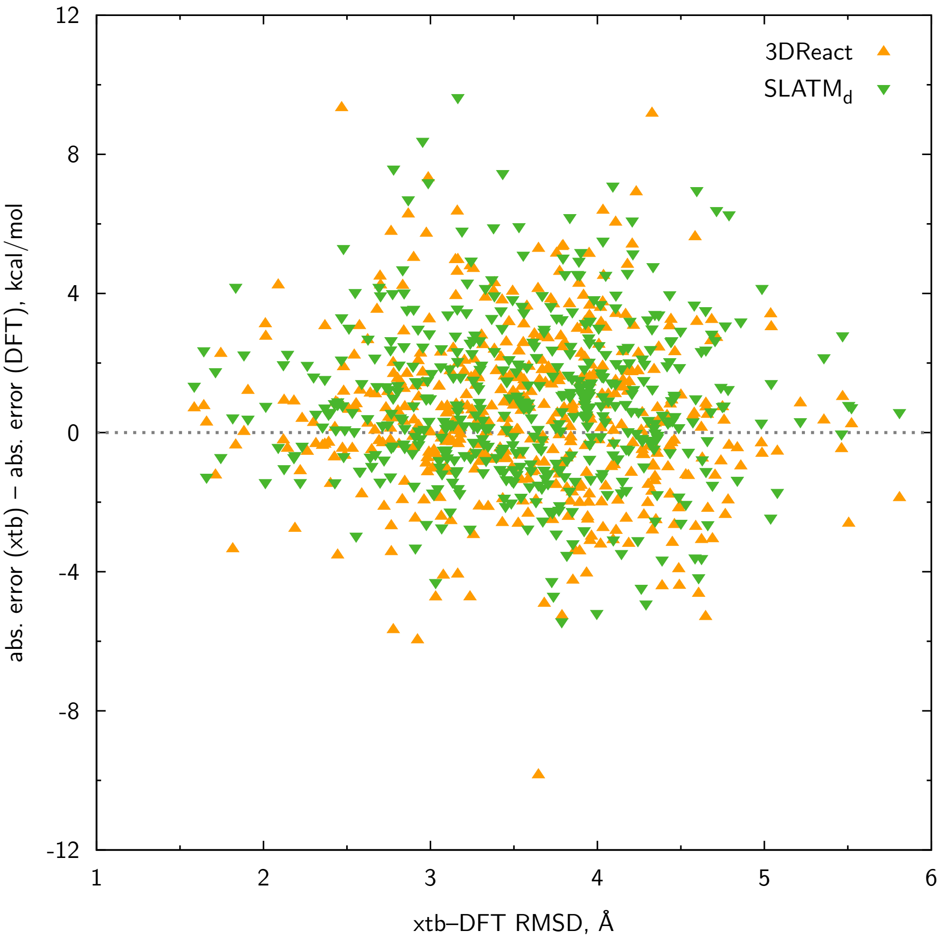

FIG. S5. Difference between absolute prediction errors using lower-quality GFN2-xTB[S12] (xtb) and provided (DFT) geometries *vs.* root-mean-square distance (RMSD) between said geometries on the Cyclo-23-TS set for 3DReact (InReact$_M$ "True") and SLATM$_d$+KRR.

S8. GEOMETRY SENSITIVITY FOR THE CYCLO-23-TS DATASET

Figure S5 shows the difference between absolute errors of models trained using GFN2-xTB (xtb) and DFT geometries for both 3DReact and SLATM$_d$+KRR *vs.* the root-mean-square distance (RMSD) between the xtb and DFT geometries (as a measure of the agreement of the structures). There is no noticeable trend for either model. This is likely because a model trained on lower quality geometries then struggles universally to predict barriers for lower quality geometries, rather than resulting in larger errors for higher RMSD molecules.

RMSD[S10] is computed as $\sqrt{\mathrm{RMSD}^2_{\mathrm{reactant}_1} + \mathrm{RMSD}^2_{\mathrm{reactant}_2} + \mathrm{RMSD}^2_{\mathrm{product}}}$ using the `rmsd`[S11] python package.

REFERENCES

[S1] G. Landrum, P. Tosco, B. Kelley, Ric, Sriniker, D. Cosgrove, Gedeck, R. Vianello, NadineSchneider, E. Kawashima, D. N, G. Jones, A. Dalke, B. Cole, M. Swain, S. Turk, AlexanderSavelyev, A. Vaucher, M. Wójcikowski, Ichiru Take, D. Probst, K. Ujihara, V. F. Scalfani, G. Godin, A. Pahl, Francois Berenger, JLVarjo, R. Walker, Jasondbiggs, and Strets123, rdkit/rdkit: 2023_03_1 (q1 2023) release (2023).

[S2] G. Corso, H. Stärk, B. Jing, R. Barzilay, and T. Jaakkola, arXiv preprint , arXiv:2210.01776 (2023).

[S3] `https://docs.e3nn.org/en/stable/guide/irreps.html`.

[S4] M. Geiger, T. Smidt, A. M., B. K. Miller, W. Boomsma, B. Dice, K. Lapchevskyi, M. Weiler, M. Tyszkiewicz, M. Uhrin, S. Batzner, D. Madisetti, J. Frellsen, N. Jung, S. Sanborn, jkh, M. Wen, J. Rackers, M. Rød, and M. Bailey, e3nn/e3nn: 2022-12-12 (2022).

[S5] A. Vaswani, N. Shazeer, N. Parmar, J. Uszkoreit, L. Jones, A. N. Gomez, Ł. Kaiser, and I. Polosukhin, Adv. Neural Inf. Process. Syst. **30**, 5998 (2017).

[S6] A. Paszke, S. Gross, F. Massa, A. Lerer, J. Bradbury, G. Chanan, T. Killeen, Z. Lin, N. Gimelshein, L. Antiga, A. Desmaison, A. Kopf, E. Yang, Z. DeVito, M. Raison, A. Tejani, S. Chilamkurthy, B. Steiner, L. Fang, J. Bai, and S. Chintala, Adv. Neural Inf. Process. Syst. **32**, 8026 (2019).

[S7] K. Spiekermann, L. Pattanaik, and W. H. Green, Sci. Data **9**, 417 (2022).

[S8] P. Schwaller, B. Hoover, J.-L. Reymond, H. Strobelt, and T. Laino, Sci. Adv. **7**, eabe4166 (2021).

[S9] B. Huang and O. A. von Lilienfeld, Nat. Chem. **12**, 945 (2020).

[S10] W. Kabsch, Acta Crystallogr. A **32**, 922 (1976).

[S11] J. C. Kromann, Calculate root-mean-square deviation (RMSD) of two molecules using rotation, `https://github.com/charnley/rmsd/releases/tag/rmsd-1.5.1` (2023).

[S12] C. Bannwarth, S. Ehlert, and S. Grimme, J. Chem. Theory Comput. **15**, 1652 (2019).
